# Genomic complexity of the variable region-containing chitin-binding proteins in amphioxus

**DOI:** 10.1186/1471-2156-9-78

**Published:** 2008-12-01

**Authors:** Larry J Dishaw, M Gail Mueller, Natasha Gwatney, John P Cannon, Robert N Haire, Ronda T Litman, Chris T Amemiya, Tatsuya Ota, Lee Rowen, Gustavo Glusman, Gary W Litman

**Affiliations:** 1 All Children's Hospital, Department of Molecular Genetics, 801 Sixth Street South, St. Petersburg, FL 33701, USA; 2 H. Lee Moffitt Cancer Center and Research Institute, 12902 Magnolia Avenue, Tampa, FL 33612, USA; 3 Department of Pediatrics, University of South Florida College of Medicine, USF/ACH Children's Research Institute, 830 First Street South, St. Petersburg, FL 33701, USA; 4 Benaroya Research Institute, 1201 Ninth Avenue, Seattle, WA 98101, USA; 5 Department of Evolutionary Studies of Biosystems, The Graduate University for Advanced Studies, Kamiyamaguchi 1560-35, Hayama 240-0193 Japan; 6 Institute for Systems Biology, 1441 N. 34th St, Seattle, WA, 98103, USA

## Abstract

**Background:**

The variable region-containing chitin-binding proteins (VCBPs) are found in protochordates and consist of two tandem immunoglobulin variable (V)-type domains and a chitin-binding domain. We previously have shown that these polymorphic genes, which primarily are expressed in the gut, exhibit characteristics of immune genes. In this report, we describe VCBP genomic organization and characterize adjacent and intervening genetic features which may influence both their polymorphism and complex transcriptional repertoire.

**Results:**

VCBP genes 1, 2, 4, and 5 are encoded in a single contiguous gene-rich chromosomal region and VCBP3 is encoded in a separate locus. The VCBPs exhibit extensive haplotype variation, including copy number variation (CNV), indel polymorphism and a markedly elevated variation in repeat type and density. In at least one haplotype, inverted repeats occur more frequently than elsewhere in the genome. Multi-animal cDNA screening, as well as transcriptional profilingusing a novel transfection system, suggests that haplotype-specific transcriptional variants may contribute to VCBP genetic diversity.

**Conclusion:**

The availability of the *Branchiostoma floridae *genome (Joint Genome Institute, Brafl1), along with BAC and PAC screening and sequencing described here, reveal that the relatively limited number of VCBP genes present in the amphioxus genome exhibit exceptionally high haplotype variation. These VCBP haplotypes contribute a diverse pool of allelic variants, which includes gene copy number variation, pseudogenes, and other polymorphisms, while contributing secondary effects on gene transcription as well.

## Background

The variable region-containing chitin-binding proteins (VCBPs), that have been identified in protochordates, represent a phylogenetically ancient example of a highly diversified family of immunoglobulin superfamily (IgSF) variable (V)-type encoding immune-type genes [1,2]. Several features of VCBPs, including the presence of two V-LIKE DOMAINS [3], distribution into characteristic subgroups, specific expression in the gut and focal regions of sequence hypervariation, are consistent with a role in immune-type recognition [4]. Hypervariation in VCBPs was described initially as a population phenomenon in which each additional study animal was observed to possess at least one allele not observed previously [2]. The extraordinarily high degree of sequence polymorphism in VCBPs is consistent with a considerable repertoire of recognition specificities [2]. Certain other features of VCBPs, including the position of the hypervariable regions, which are displaced relative to the V-DOMAINS of immunoglobulins (Ig) and T-cell receptors (TCR), as well as the apparent lack of a contiguous J region, are seemingly inconsistent with basic features of Ig and TCRs [3,5-7]. The solved structure of a VCBP reveals that the regions of sequence hypervariation map to a contiguous surface that is distinct from those involved in conventional antigen binding, recognition of viral proteins or binding of superantigens by mammalian Ig or TCR [8]. Furthermore, the structural analysis indicates that a structure emulating a J region is present. In this manuscript, sequencing and detailed annotation of the VCBP gene loci has been carried out and interpreted within the context of the first draft of the amphioxus genome (Joint Genome Institute, Brafl1). The results define the haplotypic complexity of VCBPs and identify several features of proximal chromosomal regions that could potentially relate to the high level of sequence polymorphism associated with this multigene family.

## Results

### Screening of a genome library with VCBP probes

**Figure 1:**
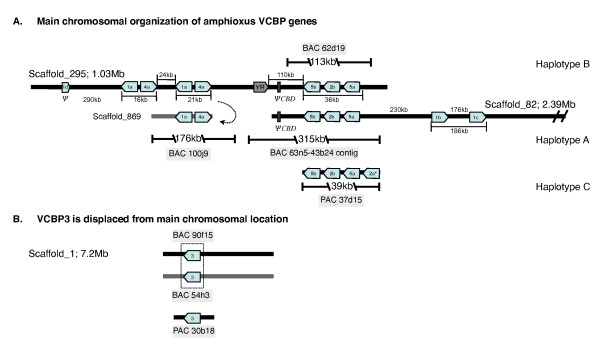
**Genomic organization of VCBP1, 2, 4, 5 and VCBP3**. A. Overlapping scaffolds_295 and 82 represent VCBP haplotypes B and A, respectively; assemblies are based on Brafl1, which was corrected from sequencing of BAC clones 62d19, 63n5-43b24 and 100J9. Information also derives from sequencing of additional BACs and PACs. VCBP1/4 is represented by a pair of artifactual gene duplicates on scaffold_295. BAC 100j9 possesses the second set of VCBP1/4 gene pairs (see Additional files 5 and 6) incorrectly assembled onto scaffold_295; correct assignment is scaffold_869 (haplotype A) (dotted arrow; authors note: assembly correction). The first set of VCBP1/4 genes on scaffold_295 is supported by direct PCR and limited DNA sequencing from BAC 5h9 (data not shown). The contiguous genetic region defined by haplotypes A (scaffold_82) and B (scaffold_295) spans ~1.5 Mb. PAC 37d15 (independent animal) represents haplotype C and possesses a VCBP2 CNV (VCBP 2a*); PAC 34i17, not shown, represents a unique VCBP1/4 haplotype. Shaded box (labeled YR) represents a Ngaro-like tyrosine recombinase retroposon gene (see text).  Pseudogenes (Y)  are indicated while intergeneic distances are in kb (1,000 base pairs) but not to scale. Transcriptional orientation is indicated by directionality of gene boxes; a and b designations reflect paralogous relationships. B.  VCBP3 is located on scaffold_1; an independent PAC allele (see Additional files 7 and 10) exhibits allelic variation. Screening of the BAC library revealed two VCBP3 alleles localized to BACs 90f15 and 54h3 (see Additional file 8). CHEF analyses (not shown) and extensive BAC library screening are inconsistent with linkage between the VCBP1/4 and 2/5 clusters and the VCBP3 gene.

In order to eliminate assembly artifacts as well as other inconsistencies noted on inspection of Brafl1 and to achieve the level of refinement required for unequivocal assignment of haplotypic variation in VCBP loci, specific reference genome BAC clones were sequenced (Table 1 and Additional file 1: Table S1 and Table S2) and assembled. The reference genome BAC sperm library was screened using probes specific for VCBPs 1, 2, 3, 4 and 5 subgroups [1]. Additional VCBP genes and alleles were recovered from a multi-animal PAC library (see Additional file 1: Table S1 and Table S2). VCBP2 and VCBP5, closely related subgroup genes, are more distantly related to VCBPs 1, 3, or 4 but share the same domain structure. VCBP5 consists of an N-terminal [D1] V domain of the VCBP2-type but possesses a distinct (VCBP5-type) C-terminal [D2] V domain and a [D3] chitin binding domain (CBD) [1]. Probes specific for VCBP2 and VCBP5 co-hybridize consistently, indicating close physical linkage (Fig. 1 and Additional file 2). Probes specific for VCBP1 and VCBP4 co-hybridized in some but not all BACs; other BAC clones hybridize only with VCBP1 probes and are not linked to BACs encoding VCBPs 1 and 4 (Fig. 1). VCBP3-specific probes did not hybridize to VCBPs 1, 2, 4 or 5-containing BACs.

**Table 1:** Haplotypic organization of VCBP genes and alleles based on BAC and PAC assisted reassembly efforts.

**Haplotype genes**	**Genomic scaffold BAC/PAC support**	**Alleles**
Haplotype A	Scaffold_82; _869	
VCBP 5a	BAC 63n5-43b24 contig	VCBP5S2*01
VCBP 2b	BAC 63n5-43b24 contig	VCBP2S1*02
VCBP 5b	BAC 63n5-43b24 contig	VCBP5S1*02
VCBP 4a	BAC 100j9	VCBP4S1*02
VCPB 1a	BAC 100j9	VCBP1S1*02
VCBP 1b	Note^1^	VCBP1S3*01
VCBP 1c		VCBP1S2*01
Haplotype B	Scaffold_295	
VCBP 5a	BAC 62d19	VCBP5S2*02
VCBP 2b	BAC 62d19	VCBP2S1*03
VCBP 5b	BAC 62d19	VCBP5S1*03
VCBP 4a	BAC 5h9	VCBP4S1*04
VCBP 1a	BAC 5h9	VCBP1S1*03
VCBP 1d	Note^2^	VCBP1S4*01
Haplotype C	Independent animal	
VCBP 5a	PAC 37d15	VCBP5S2*03
VCBP 2b	PAC 37d15	VCBP2S1*04
VCBP 5b	PAC 37d15	VCBP5S1*04
VCBP 2a	PAC 37d15	VCBP2S2*01
Haplotype A/B: VCBP3	Scaffold_1	
VCBP3	BAC 90F15	VCBP3S1*02
VCBP3	BAC 54h3	VCBP3S1*03
Haplotype X	Independent animal	
VCBP3	PAC 30b18	VCBP3S1*04

Haplotypes and gene naming are consistent with Figure 1, where a, b, c, etc. are paralogous VCBP genes. In the absence of known genetic linkage, haplotypic assignment of VCBP3 alleles is not clear, relative to the other VCBPs, see Figure 1. Haplotype X is an independent animal from the same multi-animal library from which haplotype C was acquired, yet no relationship between the haplotypes is discernable. Accession numbers and downloadable sequences are available in Additional file 1. Note^1 ^BAC support was not pursued for VCBP1b and VCBP1c. Note^2 ^VCBP1d is a five exon pseudogene. BAC support was not pursued; a VCBP5-like pseudogene consisting of only the CBD exon is also present on haplotype A and B but not listed here, see Additional file 1.

### VCBP2/5 cluster

**Figure 2:**
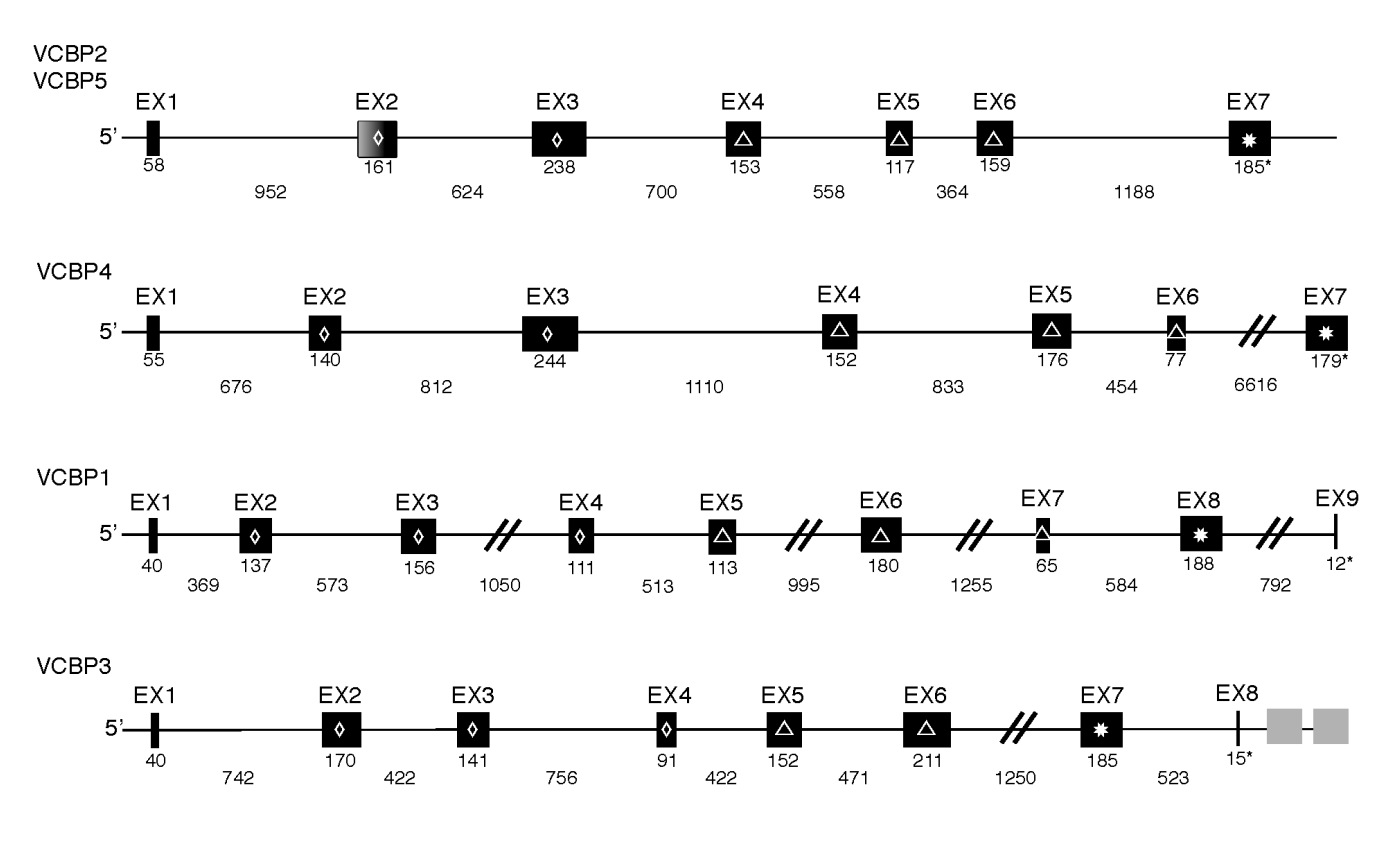
**Schematic representation of the exon/intron organization of VCBP genes**. VCBPs belong to five subgroups, each gene encodes three domains; a pair of V-LIKE DOMAIN [3], designated as [D1] and [D2] V domains, and a C-terminal chitin-binding domain (CBD). VCBP2 and VCBP5 are related closely and share the same overall exon/intron organization; the other VCBPs are more distantly related. A hyper-polymorphic region is localized to the second exon (EX2, shaded) encoding the N-terminal region of the [D1] V domain of VCBP2 and VCBP5. Additional polymorphisms have been noted (not shown) in various exons among different VCBP subgroup alleles. Exon and intron sizes are to scale or marked by (//). Lengths in nucleotides are indicated directly below exons and introns.  Secretion signals are encoded by EX1. Termination exons end in (*). All VCBP exons utilize splicing frame 0 (sf0) (Aide-memoire, Splicing sites, http://www.imgt.org/), see Additional file 1: Table S1.  One representative allele was chosen for illustration purposes, and selected if the same exon/intron characteristics were observed in at least two other alleles; alleles are:  VCBP5S1*02, VCBP4S1*02, VCBP1S1*02, and VCBP3S1*02, respectively (see Additional file 1: Table S1). VCBP3 alleles possess additional, distantly related CBD-encoding ORFs (grey boxes). Some of these appear to have splice signals and one version represents an out-of-frame splice variant. A similar sequence was recovered from the transcription products of PAC 30b18 expression in a 293T transfection assay (see text). Variation in exon sizes and extensive variation in introns exists across most alleles and is noted (see text). Domains [D] are specified as: white diamond = [D1] V, white triangle = [D2] V and white star = [D3] CBD; stop codons are indicated by (*).

**Figure 3:**
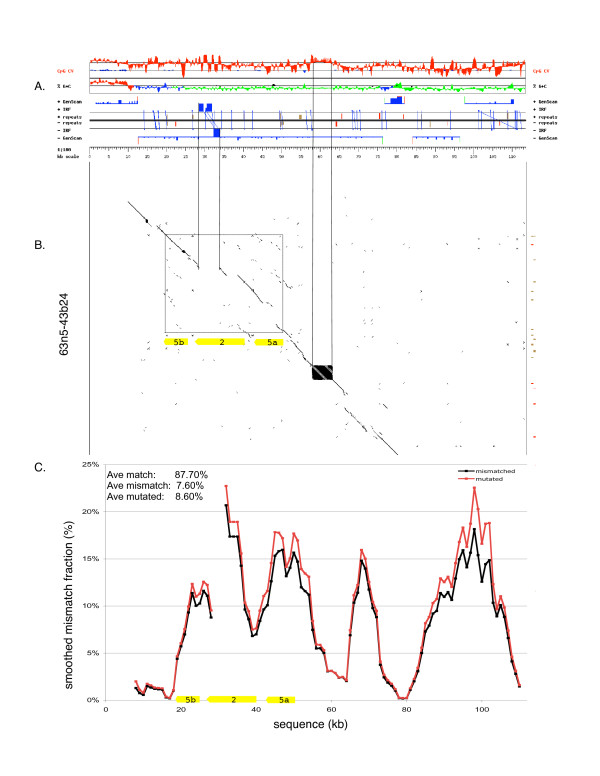
**Allelic polymorphism across the VCBP2/5 gene cluster**. A. Genomic profile (using Gestalt viewer) of BAC 62d19, indicating the location of inverted repeats (IRs) in the context of other genomic features. The top two graphs represent the CpG contrast values (observed/expected) and the G+C percentage (green – below 43%, blue – between 43% and 50%, red – above 50%), both shown as deviations from the regional average. The middle section represents features with directionality: features above the black midline are in the forward strand, while those displayed under the midline are in the reverse strand of the sequence. The various tracks depict interspersed repeats (SINEs in red, LINEs in green, DNA and LTR elements in brown, other repeats in purple), inverted repeats (IRF: blue blocks represent the repeats, diagonal lines connect the corresponding repeats on opposite strands) and gene predictions by GenScan. The bottom section is the sequence scale, in kb. B. Dot plot comparison against the corresponding region from BAC contig 63n5-43b24 (y-axis) reveals extensive haplotype variation (diagonal line breaks), much of which is associated with indel polymorphism. The VCBP2/5 cluster is boxed in B and the center repeat (darkened square in B) is oriented with the corresponding region of the Gestalt genome viewer (A). A large break is highlighted and corresponds to three large inverted repeats (dark blue) within the VCBP2 gene. Note: in order to conserve space, dot plot cropped to reflect area surrounding VCBP genes. C. Calculated polymorphism (SNP; indels count as single base change, see text) across the corresponding region in (A) and (B). The results are summarized as average match, mismatch and mutated over the entire BAC: 87.7% of the aligned nucleotides are identical between the two sequences, but only 7.6% of the aligned nucleotides are different. The discrepancy is due to the presence of gaps. Accounting for mismatched nucleotides, those in front of small gaps, and counting every larger indel (>2 bp) as a single change results in 8.6% mutated between the two sequences. Sequences are shown in same orientation as Figure 1. Matched: number of nucleotides aligned and identical; Mismatched: number of nucleotides aligned, but different; Mutated: combined mutation as insertion, deletion and point mutation where frequency of all mutation divided by sequence length is the "mutated" density.

The VCBP2/5 locus is of particular interest owing to the complex polymorphism reported for these genes [2] (see Additional file 1: Table S3 and Table S4). Conventional sequencing, restriction fragment hybridization patterns and directed PCR approaches indicate that BAC 63n5 and BAC 43b24 define an ~315 kb contig to which BAC 62d19 is allelic across the VCBP2/5 cluster, defining the first two haplotypes, A and B (Fig. 1 and Additional files 2, 3, 4). The typical organization of the VCBP2/5 cluster in the genome reference animal comprises one VCBP2 flanked by a pair of paralogous VCBP5 genes, referred to hereafter as genes 5a, 2b, and 5b, respectively (Fig. 1), and share similar exon/intron organization (Fig. 2 and Additional file 1: Table S1). This designation differentiates the first and second VCBP5 genes, which differ markedly. The VCBP2/5 gene clusters from the BACs exhibit marked haplotypic variation that extends to include miscellaneous, unrelated coding regions (see Additional files 2, 3, 4). Based on haplotypes A-C, the VCBP2/5 cluster ranges in size from 25–40 kb and exhibits inter- and intragenic variation. Large indels that include the presence/absence and/or the expansion/shrinkage of repeats and fragments of mobile elements are evident (Fig. 3 and Additional files 2, 3, 4).

This assembly is inconsistent with Brafl1 that predicts two VCBP5 pseudogenes (consisting of missing or disrupted exons), both flanking an intact VCBP2 gene. The repetitive nature of some segments of the VCBP2/5 genetic region, which includes three large flanking repeat regions (see Additional files 2, 3, 4), likely confounded the correct assembly, as reported for other genomes [9]. An additional open reading frame (ORF) encoding a VCBP5-type CBD is positioned 100 kb distal to the VCBP2/5 cluster, immediately adjacent to the middle repeat in BAC contig 63n5-43b24 (see Additional file 2). BAC contig 63n5-43b24, which represent the allelic counterpart to scaffold_295 across the VCBP2/5 gene cluster, can be assigned to scaffold_82 (2.39 mb), which spans ~100 kb of scaffold_295, extending it 2.1 mb downstream (Fig. 1 and Additional file 4).

PAC 37d15, recovered from a pooled animal library, was used to characterize a third VCBP2/5 cluster defining a new haplotype (haplotype C). Accurate assembly of this sequence required restriction fragment mapping, sequencing of PCR-generated fragments and deletion subcloning. Three (99% identical) copies of an ~4.3 kb repeat with open reading frames related to non-LTR LINE-like elements are encoded within three introns of the VCBP genes, in which a portion of an additional (upstream) VCBP2 paralog also was identified (Additional file 1: Table S1). The 37d15 haplotype arrangement is likely VCBP 2a, 5a, 2b, 5b. Although an orthologous allele of this additional VCBP2 gene (i.e., a four gene haplotype) is not present in the genome reference animal, additional copies of VCBP2 or VCBP5 genes have been noted in large scale screenings of additional animals (L. Dishaw, unpublished) and likely represent VCBP2/5 copy number variants (CNVs).

### VCBP1 and 4 genes

BAC 100j9 (VCBP1^+^/4^+^) encodes single VCBP1 and VCBP4 genes in opposing transcriptional orientation (Fig. 1). One set of VCBP1/4 genes was defined in the reference genome based on: restriction mapping, directed PCR, additional DNA sequencing of an ~4–8 kb sub-library and dot plot pairwise comparisons (Fig. 1 and Additional files 5 and 6). Restriction mapping and light sequencing of PAC 34i17 and several other VCBP1 and VCBP4 positive BACs (alternative alleles) are consistent with allelic variation across the VCBP1/4 locus (Additional file 1: Table S1). In addition to the tightly linked VCBP1/4 pair, a single pseudogene, as well as two other VCBP1 genes, are upstream and downstream to the VCBP2/5 cluster, respectively, on the same chromosomal region (Fig. 1 and Additional file 1).

BAC 100j9 (VCBP1^+^/4^+^) and 62d19 (VCBP2^+^/5^+^) span two regions of the VCBP-containing scaffold_295 (1 mb). Based on sequence data acquired here (Additional files 5 and 6), the VCBP1 and VCBP4 genes found on BAC 100j9 were misassigned to scaffold_295 as a duplicated set in the initial assembly of the amphioxus genome (Brafl1); this region of the scaffold represents the allelic counterpart (BAC 5h9). However, BAC 100j9 can be placed accurately on a short scaffold_869 that is allelic to scaffold_295 across the VCBP1/4 genetic region (Fig. 1).

### VCBP3 gene

BACs that hybridize with a probe complementing VCBP3 do not co-hybridize with probes complementing VCBPs 1, 2, 4 or 5 and thus are likely not physically linked to these latter VCBP genes (Fig. 1 and Additional files 7 and 8). The initial genome assembly indicates a single allele of a single copy gene for VCBP3 on scaffold_1, suggesting a lack of polymorphism. Restriction mapping, primer-directed PCR and limited sequencing were used to screen the VCBP3^+ ^BACs of which BAC 90f15 and BAC 54h3 are allelic. PAC 30b18 encodes a distinct third allele of VCBP3 (Additional file 1).

VCBP3 is distinguished from the other VCBP genes in its genomic organization. Specifically, [D2] V is encoded in two versus three exons as seen in the other VCBP genes (Fig. 2). Complete or partial exons, some of which encode canonical splice sites, were identified in intronic regions and exhibit extensive sequence identity to adjacent predicted coding exons (data not shown). Furthermore, two additional chitin-binding domains (CBDs) map downstream from the CBD that is proximal to the V-encoding exons (Fig. 2). The three exons encoding CBDs are divergent from each other and in some cases possess both acceptor splice and termination signal sequences. Complementary DNA (cDNA) that incorporates the third downstream CBD exon was recovered by RT-PCR using RNA isolated from haplotypically-distinct animals. The cDNA possess a premature stop codon at the CBD splice junction; however, a pair of polyadenlyation signals are encoded that would give rise to an mRNA encoding both [D1] and [D2] V domains (unpublished).

### Additional CBDs throughout the amphioxus genome

Queries of the amphioxus genome reveal a variety of genetic regions encoding CBD-related ORFs. VCBPs 2, 4 and 5 contain exons encoding type-2 version (i.e., ChtBD2) CBDs; VCBPs 1 and 3 contain exons encoding CBM_14-type CBDs and are related to those identified in peritrophins (glycoproteins associated with the chitin-rich gut environment of insects). The amphioxus genome includes >250 CBD -encoding ORFs distributed over 31 scaffolds, of which most of these are of the CBM_14-type, depending on search criteria and queries. Approximately 70 of these ORFs can be retrieved using default expect values for BLAST and the VCBP3 CBD exons as queries. The remaining CBM_14-type CBDs are related more distantly. Most CBDs are encoded by a single ORF; however, several were split in two. This latter feature is more difficult to detect and could result in a significant undercount in the actual number of CBDs. While the actual number of functional CBD exons in amphioxus is currently unknown, a variety of randomly chosen CBD-encoding ORFs revealed sequences encoding chitinase-like proteins when used as queries against the JGI gene models (Brafl1, predicted gene models). The functional relevance of the additional CBDs in amphioxus is unknown, but a variety of antimicrobial properties have now been described for CBD-containing proteins from both animals and plants [10-14].

Using the same search criteria, 12 CBDs can be identified in the genome of the cnidarian, *Nematostella vectensis *[15], whereas the sea urchin, *Strongylocentrotus purpuratus* (Echinoderm; earlier diverging deuterostome), appears to lack CBD-containing proteins altogether. Approximately 35–40 CBDs can be identified in, *Ciona intestinalis*, a urochordate that has three VCBP genes related to amphioxus VCBP3. In *Ciona*, most of the CBD-encoding DNA segments appear to be fragmented, while the full-length CBD ORFs appear to belong to, or are derived from, VCBP-related genes.

### Pseudogenes and other features of the VCBP locus

The chromosomal region encoding the VCBP2/5 haplotypes has been characterized further using various combinations of database searches (BLAST), gene prediction/modeling, and repeat masking. A high density of non-VCBP-related full-length and fragmented genes (see Additional file 1: Table S5) across the VCBP genetic region is evident and their content may vary due to large haplotype-specific indels. Relatively few VCBP pseudogenes have been identified in the amphioxus genome outside of allelic scaffold_295 or scaffold_82. A recombined, paralogous VCBP4 gene, in which the [D1] V exons are downstream of the exon encoding the CBD, is predicted from scaffold_466. A JGI-modeled transcript (Brafl1_ 104535) across this VCBP4, which also encodes a C-terminal region with four membrane-spanning units, has not been recovered using RT-PCR approaches. Likewise, a paralogous VCBP1 (JGI, Brafl_87305) can be modeled from scaffold_160. In this case, a coding region is predicted that includes two novel domains, both a death effector domain (DED)-like and death-like domain, followed by a single VCBP1-type V domain and a C-terminal CBD. It has not been possible to detect a transcript corresponding to this prediction from RT-PCR experiments using tissues from pooled animals. Based on the current assembly (Brafl1), an additional paralogous VCBP1 pseudogene, missing a portion of the coding region, is positioned upstream of the VCBP1/4 genetic region in Fig. 1. No other VCBP genes (including pseudogenes) have been identified in the draft sequence of the amphioxus genome. BAC screening did not identify any genes that were not localized in the initial draft genome assembly.

An Ngaro-like retrotransposon, which contains three ORFs coding for putative gag, reverse transcriptase/ribonuclease and tyrosine recombinase [16], is found in the vicinity of the VCBP2/5 cluster (haplotype B; see Additional file 9). Although several other Ngaro-like retrotransposons or their relics can be found distributed throughout the amphioxus genome, the arrangement of split direct repeats found in the VCBP2/5 cluster is distinct from those found in other regions of the amphioxus genome, as well as in other invertebrate genomes. Preliminary PCR data (not shown) suggests that this retroposon, unlike other features of the VCBP haplotypes (see below), is not haplotype-specific.

### Haplotype-specific variation across the VCBP2/5 gene cluster

**Figure 4:**
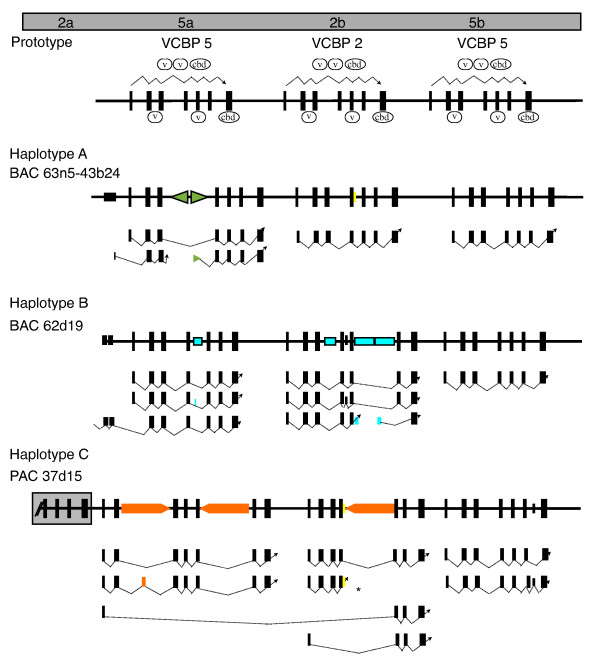
**Sequence variation and transcriptional profiles of the VCBP2/5 genes in three haplotypes**. The VCBP2/5 cluster is depicted with conserved exon (vertical bars) and intron organization (see Fig. 2); a conventional splicing pattern (dotted arrow line) is shown above the line. Predicted, as well as experimentally validated, splice variants are indicated below exon/intron organization diagrams. Haplotype-specific inserts are shown as condensed (colored) shapes between exons (green = ribonucleotide reductase gene fragments forming inverted repeats, blue = fragmented coding regions for gypsy-type mobile element and red = three identical copies of a sequence related to LINE-like elements); arrowheads are intended to represent orientation relative to paired repeat partners.

**Figure 5:**
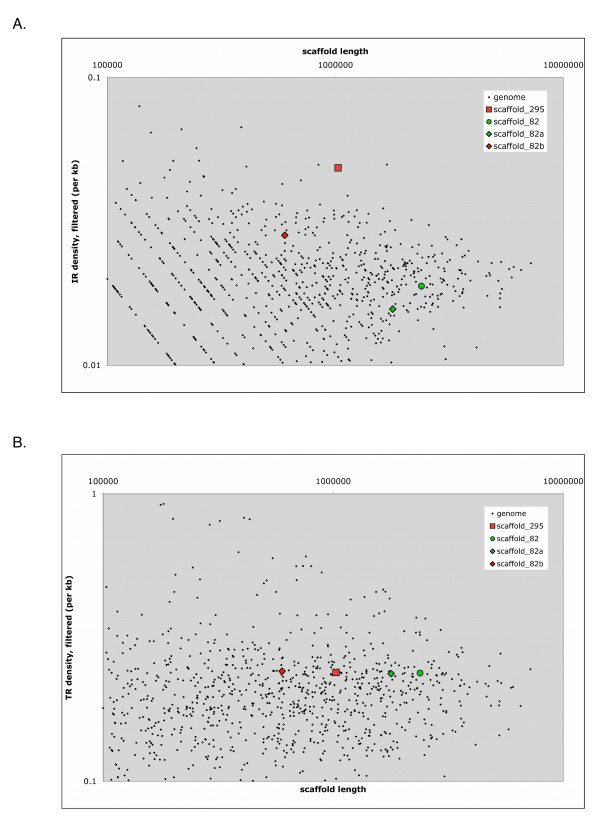
**Genome-wide comparison of repeat densities**. A. Inverted repeats (IRs) associated with VCBP2/5 clusters represented by scaffold_295 (haplotype B) and scaffold_82 (haplotype A). The density of IRs in scaffold_295 (and scaffold_82, or the corresponding region of scaffold_82 only, shown as _82b) was compared to the distribution of IRs across the entire *Branchiostoma floridae *genome. Scaffold_295 (haplotype B) possesses a higher density of IRs than most other scaffolds of related size (and larger) in the amphioxus genome, including scaffold_82 (haplotype A). A large density of the IRs is clustered in the VCBP-associated regions of scaffold _82 (_82b). B. A similar analytical approach was used to compare the distribution and density of tandem repeats. Tandem repeat density in the VCBP locus is consistent with other areas of the genome and are not found to be elevated across these two haplotypes.

As indicated previously, the VCBP2/5 cluster is of particular interest in terms of allelic variation. Pairwise sequence comparison of the BAC and PAC clones, employing dot plot analysis and pairwise alignments reveals that frequent sources of variation across the entire length of VCBP haplotypes are large (> 3 bases; up to several kilobases) and small (1–3 bases) indel polymorphisms. These can include coding regions and/or (conserved) non-coding segments (NCS), defined as fragments of sequences that are either unique to the locus (i.e., haplotype-specific) or are conserved perfectly throughout the genome (Fig. 3 and Additional files 2, 3, 4 and 10). Additional genomic PCR comparisons have revealed a variety of shared indels across haplotypes (data not shown), some of which are predicted to affect gene structure and regulation, as well transcription (Fig 4, see below). The calculated polymorphism (SNPs & indels count as single change) across the region encompassing the VCBP2/5 cluster is 8.6% (Fig. 3C), as compared to 6–7% across the genome, the highest polymorphism rate observed in sequenced metazoan genomes [15]. PCR genotyping of additional animals (data not shown), using primer sets derived from the three VCBP2/5 haplotypes described here, reveals extensive variation in genomic PCR-product size and, in some cases, absence of detectable product (L. Dishaw, unpublished). This latter effect is potentially due to either large inserts (indels) that preclude amplification or sequence variation that leads to primer incompatibility. As regards this latter point, appropriate controls were included to confirm PCR reliability. The VCBP2/5 cluster of haplotype B (scaffold_295) exhibits a higher frequency and density of inverted repeats (IRs) than is seen in the rest of the genome (Fig. 5A), whereas the density of tandem repeats is consistent with the observed genome-wide densities (Fig. 5B). The IR density of haplotype A (scaffold_82) from the genome reference animal is more consistent with the overall genome, suggesting that IR density is haplotype-dependent. This level of variation in repetitive elements could influence genomic stability of the extended VCBP gene cluster in a haplotype-specific manner.

### Haplotype-specific exchanges alter gene structure and transcriptional potential

Extensive indel variation is a feature of the VCBP gene clusters. PCR haplotyping reveals sequence variation patterns that are consistent with exchange of sequence segments from elsewhere in the genome across haplotypes. Full-length gene comparisons across the VCBP2/5 clusters of haplotypes A, B and C described here reveal diverse indels that could influence gene structure and regulation. For example, two nearly identical copies (in opposing orientations and with a short spacer region) of a single exon gene fragment orthologous to ribonucleotide reductases are present in VCBP 5a of BAC contig 63n5-43b24 (haplotype A). A hybrid transcript of ribonucleotide reductase and 3' region of VCBP 5a is predicted (Fig. 4).

Three long sequence insertions are present in VCBP 5a and 2b of BAC 62d19 (haplotype B) (see Fig. 4). One of the introns of the 5a gene includes a ~1.2 kb reverse transcriptase/endonuclease-type sequence, an intron in the VCBP 2b gene contains an ~3 kb insert and another downstream intron contains an additional ~6 kb insert. Both inserts encode small ORFs related to gypsy-type retroelement sequences. The three inserts also include pairs of large IRs (Fig. 3). This IR pair is absent in the VCBP2/5 cluster of the other two haplotypes A and C and is not found elsewhere in the genome. Exon prediction and gene modeling across the 5a gene of 62d19 predicts a typical VCBP 5a transcript and one alternative splice variant, which would lengthen the [D2] V domain through an ORF contributed by the haplotype-specific insert. Other gene models outside of the immediate boundary of the VCBP2/5 locus predict a VCBP5 transcript encoding two N-terminal short chain dehydrogenase/male sterility domains. The short chain dehydrogenase/male sterility-coding region is separated by ~3 kb immediately upstream of the VCBP cluster and potentially contributes an additional/alternative transcriptional start site. Another haplotype-specific insert, which contributes yet another ORF to one of the modeled transcripts described above for the 5a gene, is found in the same upstream region of the 63n5-43b24 haplotype (Fig. 4).

Two versions of VCBP 2b can be modeled for BAC 62d19, which includes segments of the two large haplotype-specific inserts described above. One form includes a splice variant that encodes a haplotype/insert-specific spacer between the two V domains. The other form gives rise to two additional transcripts: 1) a truncated version of VCBP 2b (two V domains) possessing an insert-specific ORF, an early termination and a poly-A signal; and 2) a transcript possessing an insert-specific (alternative) 5' start site and signal peptide spliced into a C-terminal CBD termination exon (see Fig 4).

The third haplotype of the VCBP2/5 cluster represented by PAC 37d15 (i.e., haplotype C), isolated from a separate animal, also has haplotype unique features (Fig. 4 and Additional file 10 part C). Three nearly identical copies of a large (4.3 kb) non-coding sequence with ORFs that are distantly related to non-LTR LINE-like elements have been identified. Two copies are in opposing orientations in two different introns of the 5a gene; the third copy is located in an intron of the VCBP 2b gene, in the same orientation as the second copy in 5a (Fig. 4). A typical VCBP5 transcript, as well as a second transcript containing a haplotype-specific repeat-related ORF (99 amino acids) that is spliced into the coding region of the [D1] V domain, are predicted from gene modeling. No significant transcriptional variants are predicted that incorporate fragments of the second and third copies of the repeat, which are in inverse orientation. One alternative VCBP 5b transcript is predicted that includes an atypical 65 amino acid spacer region between the [D2] V domain and the CBD. This additional ORF is derived from yet another haplotype-specific insert within that intron.

### Transcriptional potential of the VCBP gene cluster

Rare transcripts and/or alternative splice variants can be lost or under-represented in total RNA isolated from animal tissues. Human 293T cells effectively transcribe most foreign gene sequences and effect appropriate RNA splicing (e.g., based on previously characterized transcripts; Dishaw unpublished; see Methods). In order to address the potential functional consequences of haplotypic variation on the transcription of VCBPs, 293T cells were transfected with specific BAC or PAC clones; VCBP^+ ^transcripts were cloned and sequenced. PAC 37d15 is of particular interest owing to its unusual repeat content and likely status as a copy number variant (CNV) haplotype. Analysis of PCR products recovered from 293T cells transfected with PAC 37d15 reveal the predominant expression of full-length versions of VCBPs (mostly from VCBP 2b and 5b), as well as a variety of shorter, alternatively spliced variants, including sequences that integrate components of the 37d15-specific LINE-like repeat (Fig. 4). Certain variants represent splice products across two genes. In several cases, fragments of what were thought to represent non-coding sequence, including the haplotype-specific LINE-like repeat, are transcribed independently or are integrated into the VCBP transcript. Other unusual transcriptional variants, many with mis-sense mutations that are spliced out of frame, could represent "background" transcriptional noise. It is unlikely that these all represent functional molecules; however some transcription products may be involved in the regulation of expression of the VCBP genes. Additional screening will be required to verify the degree to which haplotype variation contributes to transcriptional variation.

## Discussion

### Genomic features of VCBPs

Some of the VCBP genetic regions proved unusually complex to assemble; however, two major overlapping scaffolds consisting of an ~1.5 Mb chromosomal span encoding VCBPs 2/5, 1 and 4 and a separate scaffold (without any evidence for linkage to the primary locus) encoding VCBP3 can be defined. In general terms, the VCBP genes are interspersed among a variety of unrelated genes. Genes and gene fragments are encoded across the entire length of the locus, in both directions, and within the intergenic and intronic regions of VCBPs. Additional gene fragments and non-coding regions identified within the VCBP genetic regions are distributed across the population as haplotype-specific indels.

By reference to the haplotypes defined in the BAC and PAC sequences reported here, the amphioxus genome assembly (Brafl1) [15] exhibits: allelic swaps, inversions and artifactual fragmentation (i.e., misassemblies). The accurate assembly of these haplotypes is confounded by the high density of a variety of repeat types, including microsatellites, other tandem repeats, fragments of (in some cases, complete) mobile elements, as well as a large number of IRs (see below). Assembly is confounded further by tight clustering of related paralogous VCBP genes separated from other VCBPs by large gene-rich intergenic distances (see Additional file 1: Table S5).

### Haplotype-variation includes gene CNV

The amphioxus reference genome (haplotypes A and B from a single individual) possesses three genes in the VCBP2/5 cluster in a 5, 2, and 5 pattern. Annotation of the same region from a separate haplotype in, PAC 37d15, reveals a fourth upstream tightly linked gene (fragment) of the VCBP2-type, consistent with a VCBP 2, 5, 2 and 5 cluster. Subsequent molecular genetic analyses reveal that variation in gene copy number not only is common in VCBPs of the 2 and 5 type, but also among VCBP3 genes (data not shown). These findings are consistent with previous observations that some animals possess more than the six alleles of the VCBP2/5-type [2] (and unpublished). CNVs likely are significant factors in both additional germline complexity and transcriptional diversity.

### Haplotype variation affects transcriptional potential

Across haplotypes, indel variation, mutation, mobile elements (e.g., interspersed repeats), and genetic exchanges could significantly affect gene structure (and function) by the addition and/or removal of splice sites, upstream regulatory sites, or ORFs. In order to explore this hypothesis, a simple *in vitro *assay was developed that involves analysis of transcripts from BAC or PAC templates transfected into human 293T cells. RT-PCR of the resulting transcripts reveals diverse products, including variants possessing haplotype-specific segments and others possessing in frame stop codons or frameshifts at the alternative splice junctions. Some of these haplotype-restricted transcripts may be functionally relevant. Contribution of novel ORFs from interspersed repeats, for example, has been termed "exonization," and recent evidence suggests this phenomenon can play a significant role in modifying transcriptional diversity [17-22]. The transcriptional potential of each haplotype has not been examined exhaustively; however, it is likely that haplotype-specific variation contributes to transcriptional diversity (Fig. 4) to a varying degree in the population.

### Haplotype-specific indels can affect gene structure and activity

Across haplotypes, indels can consist of large non-coding segments (NCS). These NCS can persist as single haplo-specific elements or have broad distributions throughout the genome (i.e., equivalent to typical repeat elements). Furthermore, certain NCSs, which can be conserved across some taxonomic lineages, have been shown to serve a variety of regulatory functions [23-27]. Extensive haplotype variation, in the form of large indels, could result from frequent population level genetic exchanges across haplotypes via yet undetermined mechanisms. These indels can affect gene content and expression by mobilizing, altering, contributing/removing both splice (e.g., cryptic splice signals) and start/stop signals. Haplotype-specific indels have been shown to influence gene activity, polymorphism, and overall stability of other gene loci [28-30]. If VCBPs function specifically in nonself recognition, shifts in allele frequencies potentially may factor in population-level responses to emerging pathogens [24,31,32].

### Inverted repeats and haplotype variation

Genome-wide comparisons of various repeat types indicate that the density of IRs (Fig. 5A) is higher in VCBP-related genetic regions than elsewhere in the genome. This phenomenon varies across sequence haplotypes (Fig. 5A and Additional file 10) and is supported by genomic screening with additional animals (data not shown). Furthermore, the VCBP2/5 BAC and PAC haplotypes possess unique sets of extended (large) IRs (palindromes; Additional file 10 part A-C). Palindromic sequences have been shown to induce gene amplifications [29,30] or mitotic (e.g., somatic) double-strand break-induced recombination [33]. Haplotype variation-induced structural modifications, particularly those associated with IRs or other haplotype-specific intragenic insertions, could influence transcription (i.e., pre-mRNA structural changes) and contribute to diversity and/or gene inactivation. In addition, haplotypic variation of this type is subject to cumulative effects [34,35] (Fig. 4). The presence/absence of palindromic sequences (or other IRs) across haplotypes suggests that some are genetically unstable [36,37], adding an additional layer of complexity to VCBP gene function and polymorphism.

Repetitive elements, such as IRs, can target a chromosomal region for recombination; as the density of repeats increases, the affected genetic region is associated with increasing instability [38-42]. Furthermore, haplotype-specific indel variation, promoted and/or accentuated by interspersed repetitive elements (or their relics), could drive the polymorphism of the VCBP genes by disrupting the homogenizing effects of gene conversion [43-48]. In the case of the VCBP2/5 cluster (Fig. 1), VCBP 5b is most likely homogenized by gene conversion; VCBP 2b, and to a much greater extent, VCBP 5a, are not and instead are experiencing rapid allelic divergence.

## Conclusion

Library screening, selective sequencing, and genomic PCR of amphioxus have revealed exceptionally complex VCBP haplotypes, characterized by extensive indel variation, elevated variation in repeat type and density, and CNV. Relationships between these sequences and transcription potential are suggested. Based on resolution of several haplotypes, readout of their transcriptional potential and definition of relationships between genomic content and sequence variation, it is apparent that discrete haplotype-specific features of the genome coincide with extensive allelic variation of VCBP genes. At this point it appears as if the evolution of the VCBPs parallels that described for vertebrate MHC regions [49,50] as well as the NK cell (KIR) receptors [51,52]; such effects may mirror at the heritable population level some of the characteristics that are achieved at the somatic level in vertebrates.

## Methods

### Bacterial artificial chromosome and P1 artificial chromosome library screens

Specimens of the Florida lancelet, *Branchiostoma floridae*, were collected along the coastline of Tampa Bay. Genomic DNA was isolated from pooled animals (N = 7) and a P1 artificial chromosome (PAC) library was constructed [53-55]. A bacterial artificial chromosome (BAC) library was constructed from the sperm of the single animal (BAC PAC Resources, Oakland, CA) and used as the genomic resource animal (CHORI-302) [2]. This library coverage is ~17-fold; filter arrays were screened using ^32^P-labeled probes complementing different VCBP coding regions [1,2]. Probes complementing the VCBP1,4^+ ^BAC were based on the 5' and 3' distal sequences of VCBP1,4^+ ^PAC 34i17. The insert lengths of Not I-digested PAC and BAC clones were inferred initially from CHEF analysis and ranged from ~39–55 kb for PACs and ~100–185 kb for BACs. PAC and BAC DNA [56] were restriction digested and biotin end-labeled to resolve haplotypic differences. Digested DNA was also Southern blotted and hybridized with gene-specific probes to further resolve physical relationships (Fig. 1 and Additional file 1).

### BAC/PAC sequencing

BAC and PAC genomic DNA was isolated with the NucleoBond DNA isolation kit (Clontech Laboratories/Macherey-Nagel). BAC clone 100j9 was sheared into ~3.5 kb and ~6 kb (average insert length) fractions, subcloned into the TOPO Shotgun Subcloning Kit (Invitrogen) and sequenced. Shotgun style sequencing and the Staden package (Gap4 assembly module) were employed to assemble BAC 100j9 contigs. Ambiguities and/or low coverage regions, as well as non-overlapping segments, were resolved by PCR amplification and sequencing, employing the BAC clones as templates. Genomic reference BACs and PACs were sequenced initially at JGI and the University of Oklahoma Genome Center, respectively. Owing to multiple assembly ambiguities, it was necessary to independently reassemble both BACs and PACs (see below). BAC 63n5, 43b24, 62d19 and PAC 37d15 sequences were assembled in Phrap (version 0.990329) using default parameters. The assembly was viewed and edited in Consed [57].

### Sequencing and sequence analysis

The fidelity of draft 1.0 of the amphioxus genome (Brafl1, JGI) across VCBP loci was verified from constituent BAC clones using various combinations of PCR-based approaches: BAC end sequencing, primer-directed sequencing, deletion subcloning and restriction fragment prediction strategies.

RT-PCR-recovered cDNAs and deduced polypeptide sequences were analyzed with Sequence Manipulation Suite [58], the EMBOSS package of sequence tools [59] via the web portal, , and BLAST searches [60] of locally-maintained databases. Subsequent alignments (cDNA or polypeptides) were constructed with CLUSTAL X [61]. Routine sequence comparisons as well as exon identification and gene annotation (using VCBP cDNA as queries) from genomic sequences were performed with local iterations of the BLAST package against local databases possessing complete BAC and PAC assemblies. Features of deduced polypeptide sequences were derived with BLASTP, and domains were identified via Pfam  and the NCBI conserved domain database . Genomic sequences were aligned primarily using a pairwise approach with the Needleman-Wunsch algorithm (employed locally with the Emboss package) or other global alignment parameters. Extended genomic regions were compared using a variety of dot plot approaches (Emboss package), primarily using a window size of 11.

### Genomic annotation and gene prediction

Genomic position and intron/exon annotation of the VCBP genes were determined initially using local BLAST searches with VCBP cDNAs (VCBPs 1–5, accession no. AF520472.1 (VCBP1S1*01), AF520473.1 (VCBP2S1*01), AF520474.1 (VCBP3S1*01), AF532182.1 (VCBP4S1*01), AF532183.1 (VCBP5S1*01), respectively) as queries onto BAC and PAC sequences. The VCBP gene and allele assignment is according to IMGT-ONTOLOGY rules [3]. Since an exhaustive EST library for the genome resource animal is lacking, e.g., only some and incomplete VCBPs are found [62] and because the VCBP genetic regions of the genome (Brafl1) reflect a variety of misassembly-associated artifacts that contribute to incorrect gene modeling, the VCBP genes described in this manuscript were computationally predicted from the supporting BAC and PAC clones. These predicted transcripts (see Additional file 1: Table S1) were compared to our extensive in-house cDNA collection, and, when possible, validated with cDNA recovered from multi-animal screenings. These and any additional genes from the BAC and PAC clones were predicted and compared first using BLASTX (NCBI) and then by creating gene models for BLASTP searches using: GenScan [63], GeneID [64,65], FGENESH and FGENESH+  or genomescan [63,66]. Details of splicing were visualized using Splign [67]. Additional genomic annotation and the GESTALT maps from Fig. 3 (and Additional file 10) were created via the GESTALT workbench [68].

### Genetic polymorphism and repeat analysis

Several categories of repetitive DNA were identified initially using RepeatMasker  and the *einverted *program of the Emboss package. Since the public repeat library for Branchiostoma is very limited (library release 20061006, including 176 ancestral and ubiquitous sequences and just 11 lineage-specific sequences), the amphioxus genome was analyzed using RepeatModeler Open-1.0.3 , a de novo repeat family identification and modeling package. This resulted in the definition of an uncurated, extensive library of putative amphioxus repeats. While the previous public library masked just 2.7% of the amphioxus genome (in 0.24 million segments), the new repeat library identifies 25.7% of the amphioxus genome as apparently repetitive (in 1.34 million segments). We consider this extended library as better suited for analysis, as it will minimize false positives from uncharacterized interspersed repeats.

A preliminary round of comparative analyses was accomplished first using ten randomly selected *Branchiostoma *BAC clones deposited in the NCBI database and later extended to the whole genome assembly. More thorough genome-wide comparisons where made with Tandem Repeat Finder  and Inverted Repeat Finder  software packages.

The density of polymorphisms along the VCBP locus was mapped using FastA [69] to align the sequences between annotated interspersed repeats in one sequence, to the complete locus in the other sequence. Repeats were identified using RepeatMasker version open-3.1.9, RM database version 20071204; corresponding repeats between the two sequences were identified by matching genomic sequences between consecutive alignments, and the genomic alignments were completed by aligning the matched repeats. In this way, aligning similar, but irrelevant repeats, was avoided. Once two genomic sequences were aligned, indels and mismatches in all aligned segments (counting indels longer than two bp as single events) were tallied into overlapping 5 kb windows, with a window jump of 1 kb. Mutation counts (mismatches plus indels) were normalized by the actual amount of aligned sequence in each window.

The genome-wide repeat density for various repeat types was estimated by analyzing the 3,032 scaffolds in the amphioxus genome draft (Brafl1) using the Inverted Repeats Finder software [70] with the following parameters: match = 2, mismatch = 3, delta = 5, match probability = 80, indel probability = 10, minscore = 10, maxlength = 10000, maxloop = 10000, and -l to exclude lowercase interspersed repeats from k-tuple matches. The resulting compilations of inverted repeats (IRs) were consolidated by filtering out alternative, overlapping IRs; those with higher scores were retained. The amphioxus genome (Brafl1) was analyzed in a similar manner using the Tandem Repeats Finder software [71]. Parameters are: match = 2, mismatch = 7, delta = 7, match probability = 80, indel probability = 10, minscore = 50, and maxperiod = 500. The resulting lists of tandem repeats (TRs) were considered by filtering out alternative, overlapping TRs, keeping those with longer word sizes. For each genomic scaffold, the total numbers of observed IRs and TRs and the scaffold length were tallied. Since the last 600 kb of scaffold_82 overlaps the region of interest, scaffold_82 was split into two parts (scaffold_82a and scaffold_82b) and the IRs and TRs were tallied separately. The IR and TR analyses were performed after masking the amphioxus genome with first the limited RepeatMasker library and then with the extended RepeatModeler library. A comparison of the two sets of alternative results showed no discrepancy when studying TRs, and more significant results when studying IRs on the RepeatModeler-masked genome sequence. These results highlight the importance of appropriate masking of interspersed repeats prior to downstream analyses.

### BAC and PAC expression in HEK 293T cells

To help predict the transcriptional potential of VCBP genetic regions, amphioxus-specific PAC or BAC DNA clones were selected and expressed in stable lines of human embryonic kidney (HEK) 293T cells. PAC clone 37d15 encodes a particularly unusual haplotype and was used to characterize transcriptional repertoires. Human HEK 293T cells were grown to 95% confluence on 10 cm plates and transfected (10–30 ug per 10 cm plate) with unmodified NucleoBond-purified (Clontech) BAC or PAC DNA using Lipofectamine 2000 (Invitrogen). After 72 hrs, total RNA was isolated from the cultures, treated with DNase, reverse transcribed and amplified using primers to known genetic regions. Primers were anchored in either the VCBP chitin-binding domain (CBD) or at the poly-A tail (e.g., oligo-dT primer) and at the upstream signal peptide-encoding region (forward or sense primer). PCR products were size-verified and subsequently cloned by direct-PCR cloning. Nylon lifts of recombinant bacterial clones, hybridized with ^32^P-labeled VCBP-specific oligonucleotides, were size-selected by PCR, and sequenced.

## Authors' contributions

LJD was responsible for overall management of the project and conducting PCR mapping, cloning, sequence analysis, contig and BAC assembly, bioinformatics, data analysis and interpretation, and prepared the manuscript. MGM was responsible for library screening, PCR cloning, sequencing, and data analysis and interpretation. NG participated in PCR, subcloning, and data analysis. JPC and RNH provided original sequences, libraries and data, and participated in sequence analysis, data interpretation and drafting the manuscript. RTL participated in sequencing, management, and data interpretation. CTA constructed the PAC library, and was involved in sequence compilation, data interpretation, and writing the manuscript. TO performed sequence analysis, bioinformatics, and data interpretation. LR assembled some of the BAC and PAC clone sequences, assisted in data analysis and interpretation, and helped draft the manuscript. GG wrote and implemented Perl scripts, performed BAC- and PAC-specific and genome-wide analysis of repetitive sequences and provided data analysis and interpretation as well as helped draft the manuscript. GWL directed the research, assisted in data analysis, and helped draft the manuscript. Research grant awards to his laboratory funded the research. All authors have read and approve of the manuscript.

## Supplementary Material

Additional file 1 **Tabular description of BAC and PAC sequences, VCBP pairwise comparisons, and annotation.** Table S1. Characterization of new VCBP alleles from genomic resource animal and additional animal PAC library-derived clones. Table S2. BAC and PAC clones used to aid in the genomic description, annotation, and validation of the VCBP genomic region of Brafl1. Table S3. Pairwise comparison (identity | similarity percentages) of deduced amino acid sequences for the VCBPs that are supported by BAC and PAC evidence. Table S4. Pairwise nucleotide identity among all alleles described in Figure 1. Table S5. Annotation of coding regions and predicted genes from the VCBP-containing BAC and PAC clones described. Complete list of transcripts is included. Click here for file

Additional file 2 **Dot plot pairwise comparison of the VCBP2/5 cluster containing BAC clones, 62d19 and 63n5-43b24 contig (haplotype B and A, respectively), reveals extensive polymorphism.** Click here for file

Additional file 3 **Dot plot pairwise comparison of BAC 62d19 and BAC contig 63n5-43b24 exhibits discontinuities associated with allelic polymorphism, notably across the region encoding the VCBP2/5 cluster.** Click here for file

Additional file 4 **Dot plot pairwise comparison of the reverse complement of BAC 62d19 and BAC contig 63n5-43b24, and the corresponding region of the amphioxus genome.** Click here for file

Additional file 5 **Dot plot pairwise comparison of the reverse complement of the VCBP1/4-containing BAC 100j9 with the corresponding region of scaffold_295.** Click here for file

Additional file 6 **Dot plot pairwise comparisons of scaffold_295 and scaffold_869 with the reverse complement of BAC 100j9 and PAC 34i7 (independent animal haplotype).** Click here for file

Additional file 7 **Dot plot pairwise comparisons of the reverse complement of a ~100 kb region of scaffold_1 encoding VCBP3 with the corresponding region from BAC 90f15 and BAC 54h3, as well as PAC 30b18 (independent animal haplotype) encoding VCBP3.** Click here for file

Additional file 8 **Dot plot comparisons of the VCBP3 gene region reveals that BAC 90f15 corresponds to genomic scaffold_1 (A) and that the other allele is highly polymorphic (B).** Click here for file

Additional file 9 **Genomic organization of the tyrosine recombinase domain-encoding retroelement found adjacent to the VCBP2/5 cluster.** Click here for file

Additional file 10 **Genomic representation (using the Gestalt viewer) across the BAC and PAC alleles described in this study.** Click here for file
