# Supplementary material for: Genomic complexity of the variable region-containing chitin-binding proteins in amphioxus
Source: BMC Genet. 2008 Dec 1;9:78. doi: 10.1186/1471-2156-9-78 (PMC2632668; doi:10.1186/1471-2156-9-78)
Supplement: Additional file 1 — Tabular description of BAC and PAC sequences, VCBP pairwise comparisons, and annotation. Table S1. Characterization of new VCBP alleles from genomic resource animal and additional animal PAC library-derived clones. Table S2. BAC and PAC clones used to aid in the genomic description, annotation, and validation of the VCBP genomic region of Brafl1. Table S3. Pairwise comparison (identity | similarity percentages) of deduced amino acid sequences for the VCBPs that are supported by BAC and PAC evidence. Table S4. Pairwise nucleotide identity among all alleles described in Figure 1. Table S5. Annotation of coding regions and predicted genes from the VCBP-containing BAC and PAC clones described. Complete list of transcripts is included. [file 1471-2156-9-78-S1.pdf]

Genomic [1]	Allele	Functionality	Exons [2]	Exon sizes	Molecule type [3]	Accession numbers
**BAC_63n5-43b24 contig**	VCBP5S1*02	F	EX1-7	M58, 161, 238, 153, 117, 159, 185*	cDNA	BK006758
	VCBP2S1*02	F	EX1-7	M46, 167, 235, 165, 120, 165, 185*	cDNA	BK006760
	VCBP5S2*01	F	EX1-7	M58, 161, 232, 153, 114, 153, 185*	cDNA	BK006759
	VCBP5S3*01	P	EX7	M185*	gDNA	pending[8]
**BAC_62d19**	VCBP5S1*03	F	EX1-7	M58, 161, 238, 153, 117, 159, 185*	cDNA	BK006763
	VCBP2S1*03	F	EX1-7	M46, 161, 241, 165, 120, 165, 185*	cDNA	BK006762
	VCBP5S2*02	F	EX1-7	M53, 155, 229, 153, 114, 156, 185*	cDNA	BK006761
**PAC_37d15**	VCBP5S1*04	F	EX1-7	M58, 161, 238, 153, 117, 159, 185*	cDNA	BK006769
	VCBP2S1*04	F	EX1-7	M46, 167, 235, 207, 120, 165, 185*	cDNA	BK006768
	VCBP5S2*03	F	EX1-7	M58, 161, 226, 150, 114, 180, 185*	cDNA	BK006755
	VCBP2S2*01	ORF[4]	EX4-7	M167, 120, 153, 179*	gDNA	
**BAC_90f15 Scaffold_1**	VCBP3S1*02	F	EX1-8	M40, 170, 141, 91, 152, 211, 185, 15*	cDNA	BK006764
**BAC_54h3**	VCBP3S1*03	F[5]	EX1, EX3-8	M40, 141, 50, 175, 211, 185, 15*	cDNA	BK006765
**PAC_30b18**	VCBP3S1*04	F	EX1-8	M40, 170, 141, 91, 152, 211, 185, 15*	cDNA	BK006766
**BAC_100J9**	VCBP4S1*02	F	EX1-7	M55, 140, 244, 152, 176, 77, 179*	cDNA	BK006757
	VCBP1S1*02	F	EX1-9	M40, 137, 156, 111, 113, 180, 65, 188, 12*	cDNA	BK006756
**Scaffold_295 BAC-5h9**	VCBP4S1*03	F[6]	EX1-4, EX6-7	M55, 140, 244, 152, 67, 179*	cDNA	pending[8]
	VCBP1S1*03	F	EX1-9	M40, 137, 156, 97, 127, 180, 65, 188, 12*	cDNA	pending[8]
**PAC_34i7**	VCBP4S1*04	F	EX1-7	M55, 140, 244, 152, 176, 72, 179*	cDNA	BK006767
	VCBP1S1*04	ORF[7]	EX1-5	M40, 137, 156, 97, 125	gDNA	
**Scaffold_82**	VCBP1S2*01	F	EX1-10	M40, 59, 24, 156, 121, 125, 182, 89, 188, 102*	cDNA	pending[8]
	VCBP1S3*01	F	EX1-9	M46, 137, 156, 97, 125, 182, 65, 188, 204*	cDNA	pending[8]
**Scaffold_295**	VCBP1S4*01	P	EX2,4,5,8	EX5 is fragmented; EX1,3,6,7,9 missing	gDNA	

Additional file 1: Table S1. Characterization of new VCBP alleles. Genomic DNA from both the genome resource animal (JGI_Brafl1), Scaffold_295 and Scaffold_82, along with supporting BAC clones, as well as PAC clones representing additional haplotypes, were used to model and predict new allelic variants of previously characterized full length VCBP cDNAs. All predicted transcripts were modeled with fgenesh+ using full-length VCBP cDNAs (AF520472, AF520473, AF520474, AF532182, and AF532183) as training queries. Splice sites, splicing frames, and exon types were visualized and confirmed with Splign (see Methods). F: functional; P: pseudogene; ORF: open reading frame.

1 Accession numbers to BAC and PAC clones are listed in Additional file 1: Table S2.

2 All exons of the VCBPs are of the M-type, using splicing frame 0 (Sf0).

3 All transcripts are predicted only. Representative cDNAs from genomic resource animal are lacking.

4 This VCBP2 allele appears to be disrupted by cloning.

5 This VCBP3 allele has a disrupted (with stop codon) second exon, but a putative alternative transcript is predicted by fgenesh(+).

6 This allele has a missing exon 5 (confirmed with BAC 5h9) but a putative alternative transcript is predicted by fgenesh(+).

7 This VCBP1 allele appears to be disrupted by cloning.

8 Accession numbers pending; Third Party Annotation (TPA) database (NCBI). BAC/PAC support lacking; sequences are attached for download.

BAC/PAC clone	Accession Number	Positive for VCBPs	Size (kb)	Sequenced	Notes
**BAC-63n5**	EU875590	2 and 5	153	Phase 3 finished	63n5 and 43b24 are same allele, overlap by 6kb, form 315kb contig; corresponds to the allele represented by scaffold_82.
**BAC-43b24**	EU875590	2 and 5	168	Phase 3 finished	
**BAC-62d19**	EU875589	2 and 5	113	Phase 3 finished	Allelic counterpart to 63n5-43b24 contig; represents scaffold_295 allele
**PAC-37d15**	AC135603.5	2 and 5	39	Phase 3 finished	Represents a third allele to the VCBP 2/5 gene cluster; misc. animal.
**PAC-30b18**	AC135602.14	3	57	2 ordered contigs	Represents a third allele to the VCBP 3 gene; represented by one allele in Braf1 in scaffold_1.
**BAC-90f15**	EU875591	3	196	partial; 10.7kb	90f15 represents allele from scaffold_1; 90f15 and 54h3 are alleles; only partial sequence as one assembled contig over VCBP3 gene region; used PCR and primer directed approach. Accession number reflects the 10.7kb sequenced and assembled contig.
**BAC-54h3**	EU875588	3	108	partial; 8.8kb	Accession number reflects the 8.8kb sequenced and assembled contig.
**PAC-34i17**	AC141443.12	1 and 4	33	Phase 3 finished	Represents a thirld allele to the VCBP1/4 cluster
**BAC-100j9**	EU875592	1 and 4	176	2 ordered contigs	Allelic counterpart to scaffold_295; represented by scaffold_869 (VCBP genes are misassembled onto scaffold_295 as a paralogous set, see text for details and Supplement Figure S4-S5).

Additional File 1: Table S2. BAC and PAC clones used to aid in the genomic description, annotation, and validation of the VCBP genomic region of Brafl1. All BAC clones from this manuscript represent the same reference animal as the JGI genome, Brafl1. PAC clones are from alternate animals and represent additional haplotypes. BAC 5h9 (not shown; only partially sequenced across gene region) is equivalent to scaffold_295 over the VCBP1/4 region and allelic to BAC 100j9. NOTE: PAC 34i17 is incorrectly labeled as 34i7 in AC141443.12.

A.

VCBP 2/5		62d19 -5a	63n5-43b24 -5a	37d15 -5a	62d19 -2b	63n5-43b24 -2b	37d15 -2b	62d19 -5b	63n5-43b24 -5b	37d15 -5b
VCBP 5a	**62d19**	100								
	**63n5-43b24**	79 | 83	100							
	**37d15**	68 | 77	69 | 76	100						
VCBP 2b	**62d19**	49 | 59	51 | 60	52 | 61	100					
	**63n5-43b24**	48 | 58	51 | 60	51 | 61	80 | 84	100				
	**37d15**	46 | 56	49 | 58	49 | 58	78 | 81	93 | 95	100			
VCBP 5b	**62d19**	63 | 73	66 | 74	71 | 77	56 | 65	56 | 65	55 |63	100		
	**63n5-43b24**	63 | 74	66 | 75	71 | 78	57 | 66	57 | 66	55 | 63	93 | 95	100	
	**37d15**	64 | 73	66 | 74	71 | 78	57 | 66	56 | 65	56 | 64	95 | 96	93 | 95	100

B.

VCBP 4	Scaff_295	BAC 100j9	PAC 34i17
**Scaffold_295**	100		
**BAC 100j9**	99 | 99	100	
**PAC 34i17**	95 | 96	95 | 96	100
**VCBP 1**			
**BAC 100j9**	96 | 98	100	N/A

C.

VCBP 3	Scaffold_1	BAC 54h3	PAC 30b18
**Scaffold_1**	100		
**BAC 54h3**	89 | 91	100	
**PAC 30b18**	94 | 95	88 | 91	100

Additional file 1: Table S3. Pairwise comparison (identity | similarity percentages) of deduced amino acid sequences for the VCBPs that are supported by BAC and PAC evidence. Full length sequences were predicted from the BAC and PAC sequences using a combination of Blast comparisons and gene modeling with *fgenesh* and *genomescan*. A. comparison of the VCBP 2 and 5 alleles (as 5a, 2b, and 5b) from the 2/5 cluster. Only BAC and PAC (as the third allele) are compared; genomic scaffolds in this region possess misassembly artifacts. Interparalogous comparisons are done because of the close relationship between these two genes. B. comparison of VCBP4 alleles. BAC 100j9 (mostly represented by scaffold_869) is allelic to scaffold_295 across the VCBP1/4 region. PAC 34i17 is the third allele, which is disrupted across the VCBP 1 gene and therefore could not be used in the VCBP1 pairwise comparison. C. comparison of VCBP 3 alleles; BAC 54h3 is allelic to scaffold_1 VCBP 3 gene and PAC 30b18 is a third allele.

A.

VCBP2/5	VCBP5S1*02	VCBP2S1*02	VCBP5S2*01	VCBP5S1*03	VCBP2S1*03	VCBP5S2*02	VCBP5S1*04	VCBP2S1*04	VCBP5S2*03
**VCBP5S1*02**	100								
**VCBP2S1*02**	73	100							
**VCBP5S2*01**	80	73	100						
**VCBP5S1*03**	94	72	80	100					
**VCBP2S1*03**	73	92	72	73	100				
**VCBP5S2*02**	79	72	88	80	71	100			
**VCBP5S1*04**	94	74	80	94	74	81	100		
**VCBP2S1*04**	72	97	72	73	92	72	74	100	
**VCBP5S2*03**	82	71	83	82	71	82	81	70	100

B.

VCBP1	VCBP1S1*02	VCBP1S1*03	VCBP1S2*01	VCBP1S3*01
**VCBP1S1*02**	100			
**VCBP1S1*03**	96	100		
**VCBP1S2*01**	86	87	100	
**VCBP1S3*01**	91	90	89	100

C.

VCBP4	VCBP4S1*02	VCBP4S1*03	VCBP4S1*04
**VCBP4S1*02**	100		
**VCBP4S1*03**	92	100	
**VCBP4S1*04**	96	93	100

D.

VCBP3	VCBP3S1*02	VCBP3S1*03	VCBP3S1*04
**VCBP3S1*02**	100		
**VCBP3S1*03**	88	100	
**VCBP3S1*04**	94	89	100

Additional file 1: Table S4. Pairwise DNA comparison of the VCBP alleles described in the manuscript. All alleles are predicted from the genomic DNA. Transcripts were predicted as described in the Methods (see Table 1 and Additional file 1: Table S1).

Additional file 1: Table S5. Annotation of coding regions and predicted genes from the VCBP containing BAC and PAC clones described. Numbers represent gene location & span; orientation of gene is implied by number direction. VCBPs are listed first; other genes follow. [1] VCBP5a and 2 are interrupted by 3 copies of a large interspersed element and alleles of 5a may incorporate element-derived ORFs. [2] Non-coding interspersed element; three copies are nearly identical (see text). Identity/similarity and e values are approximations; varies with sequence availability.

BAC/PAC clone/contigs	Gene-spanning region	Similar to known/predicted gene products	% identity | similarity	e value	comments
PAC 37d15	245…3278	VCBP 2 (VCBP2S2*01)			(1/2 gene -from cloning)
(39kb)	5262…19006	VCBP 5 (VCBP5S2*03) [1]	75 | 82	5E-145	2 copies of 4.3kb repeat (-/+)
Other animal	21821…30983	VCBP 2 (VCBP2S1*04)	72 | 79	2E-142	1 copy of 4.3kb repeat (+)
Haplotype C	32956…37990	VCBP 5 (VCBP5S1*04)	74 | 77	4E-167	
	10763…6366	4.3kb NCE;repeat [2]			
	12281…16894	4.3kb same repeat			
	24482…28880	4.3kb same repeat			
BAC 63n5-43b24	135804…145469	VCBP 5 (VCBP5S2*01)	71 | 79	6E-126	interspersed pseudogene
(315kb)	148776…154132	VCBP 2 (VCBP2S1*02)	75 | 83	2E-143	
JGI Haplotype	156065…161233	VCBP 5 (VCBP5S1*02)	95 | 97	0.0	
Haplotype A					
	43402…16590	TRAIL-like	30 | 45	1E-07	
	53501…54493	Hypothetical LRR protein	31 | 44	7E-09	
	75515…71245	Mitochondrial translocase-like partial	59 | 71	3E-17	
	84558…79306	RVT; endonuclease-like	39 | 55	6E-96	

	87776…97580	ADP-ribosylation factor interacting protein 1-like	50 | 65	7E-73	
	105293…102432	Ret tyrosine kinase receptor-like; FGF receptor 3-like	47 | 66	1E-44	
	107306…117139	Short chain dehydrogenase-like; male sterility protein-like	42 | 64	4E-53	
	117698…118954	RVT-like (different than above)	31 | 51	4E-46	
	139485…139069	Ribonucleotide reductase -partial	80 | 87	3E-59	
	140979…141395	Ribonucleotide reductase -partial	80 | 87	3E-59	
	172911…166980	Plasminogen-like; transmembrane protease	50 | 65	7E-70	
	229647…192975	Htra3 protein; serine protease	25 | 50	1E-16	
	231498…230254	Transposase-like fragment	36 | 55	6E-11	
	244637…238600	Alpha-galactosidase B-like	52 | 66	1E-127	
	245507…271539	Oral-facial-digital syndrome 1; Ofd1-like protein (partial)	36 | 56	9E-26	
	277960…274394	Tyrosine Recombinase RVT	44 | 63	6E-81	
	301091…300633	RVT fragment	38 | 59	4E-19	
	313995…311637	L-type amino acid transporter 1-like	45 | 66	1E-11	
BAC 62d19	63018…70481	VCBP 5 (VCBP5S2*02)	66 | 76	1E-123	
(113kb)	73607…86490	VCBP 2 (VCBP2S1*03)	76 | 83	2E-148	contains interspersed element
JGI Haplotype	88525…94237	VCBP 5 (VCBP5S1*03)	95 | 97	1E-179	
Haplotype B					
	10505…7674	Mitochondrial membrane protein-like	59 | 71	3E-17	
	19079…28604	ADP-ribosylation factor interacting protein 1	49 | 62	7E-106	
	35224…32369	Ret tyrosine-kinase receptor	47 | 66	1E-44	

	36958…59793	Short chain dehydrogenase; male sterility domain 2 protein	52 | 76	4E-33	
	111865…101864	Star fish protease-like; Plasminogen-like; transmembrane protease	49 | 67	4E-52	
PAC 30b18	36352…47466	VCBP 3 (VCBP3S1*04)	95 | 97	0.0	
(57.4kb)					
Other animal	20293…2170	Similar to tudor domain proteins	29 | 44	5E-28	
	23510…27344	Unknown; contains Mid-1-related chloride channel 1-like domain	35 | 52 Mid-1 domain	4E-14	
	31939…29549	similar to Urchin RVT	51 | 65	4E-77	
	56877…51842	Similar to zebrafish and human upstream binding transcription factor, RNA Pol I	37 |57	5E-41	
BAC 100j9	133287…121773	VCBP 4 (VCBP4S1*02)	83 | 88	3E-167	
(176kb)	137590…142796	VCBP 1 (VCBP1S1*02)	97 | 98	0.0	
JGI Haplotype					
Haplotype A	63159…40792	Similar to Tetraodon protein; contains SLAIN motifs	77 | 92 motifs only	variable	
	71152…72681	Similar to homeobox transcription factor	31 | 48	8E-07	
	92750…78019	Similar to TBC1 protein (GRAM domain)	55 | 72	0.0	
	94810…111515	Similar to activating signal cointegrator 1 complex	36 | 51	3E-74	
	151400…153826	Unknown; contains LRR repeats; Drosophila GF20961	36 | 52	2E-13	
	170231…167852	Similar to x globin [Strongylocentrotus purpuratus]	26 | 50	4E-11	

PAC 34i17	19871…25710	VCBP 4 (VCBP4S1*04)	84 | 89	2E-170	
(33.4kb)	29669…33400+	VCBP 1 (VCBP1S1*04)	95 | 97	0.0	
Other animal					

>VCBP1S1*03 [organism=Branchiostoma floridae] [molecule=DNA] [moltype=genomic] [location=genomic] [note=allele 03 of VCBP1; predicted transcript, BAC5h9, Scaffold_295; Brafl01 genome resource animal] Amphioxus variable region-containing chitin-binding protein 1 (VCBP1), VCBP1S1*03, mRNA; VCBP1 allele (predicted)
ATGAAGTTTGTACTTGGCTTGGTCCTTCTTGCTGTTGGCGCGCACGCCATGACCATCGTG
ACCGTCAGTACCCCTGAACCGAAGGTTGAGGCTAGTGTGGGGGGTTCCGCGGAACTCAAG
TGCGAGTTTGACATCCAACCCAACTCTACACAGCCTCCTACTATCGCCTGGTTCAAGGGC
AATGACGACTTCCGCGGTGCCGAGCGGATCTACACGGGACACAAGGTGTGGGGGAACGAG
ACGCAACGACGGGAGGACAGCTTCGGGGACTACATCGGGCGGGTGGAGGTGGCGGATCTG
GACAAACCCGCCATCAAGATCAGCGGCATCAAGAGTACCGACTTCGCGCGCTACTGGTGT
ACTGTAGCGGAGTGGGGTGTGCGGACGGAGTTCGGAGTGGACGCCAAGTCAGTTCTGCTG
ACTGAAACTGGTGAGCTTAAATCGTCCATTGACATTTCCGTTTCCGGTGAGAAGGACGTG
GATGAAGGTGGTGACGTAGAGATGACGTGTCGTTGCCATGGCTGCACTTCTGCCGCGATA
TTCGACTGGTTCAAGGTGGCGTTTGCCGGAAGTGAGTGGGTGACAACCGGAAACTACACT
CATATCGCAGCCAAGGTCGACGTTGGCGTTTTGGGATTTCCAAACCCGATAGAGATTGAC
GATGGATTTGGCCAGTTCAGCGTGACACCATCCAATTCCCTCCGTCTGACCGGGGCGCAG
GTAGCCGACGCAGGAAGGTACTGGTGTAAGGTGACAAGCGGTGGGAGCGTGGACATCAAG
GCAACCGTGCTGAAGGTCAAAGTGCCGGAGTTCACCTGTGCCGGTAAGGCTGACGGGCAC
TACCCTGACCCGGAGGACTGCGCCATGTACTACCAGTGTCTGTACGGCTTCCCTCAGCCC
TTCCACCGCCCGTGTGGGTACGCCGGCATGGTCTTCAACCCCGAGCACCTGTACTGCGAC
TGGGCCTTCAACGTGGGACCGCCATGCGGGAGCAAGGCTTAG
>VCBP1S1*02 [organism=Branchiostoma floridae] [molecule=DNA] [moltype=genomic] [location=genomic] [note=allele 02 of VCBP1; predicted transcript, BAC100J9 (EU875592), Scaffold_295; Brafl01 genome resource animal] Amphioxus variable region-containing chitin-binding protein 1 (VCBP1), VCBP1S1*02, mRNA; VCBP1 allele (predicted)
ATGAAGTTGATACTTGGCTTGGTCGTTTTTGCTGTTGGCGCGCACGCCATGACCATCGTG
ACCGTCAGTACCCCTGAACCGAAGGTCGAGGCTAGTGTGGGGGGTTCCGCGGAACTCAAG
TGCGAGTTTGACATCCAACCCAACTCTACACAGCCTCCTACTGTCGCCTGGTTCAAGGGC
AATGACGACTTCCGCGGTGCCGAGCGGATCTACACGGGACACAAGGTGTGGGGGAACGAG
ACGCAACGACGGGAGGACAGCTTCGGGGACTACATCGGGCGGGTGGAGGTGGCGGACCTG
GACAAACCCGCGATCAAGATCAGCGGCATCAAGAGTACCGACTTCGCGCGTTACTGGTGT
ACCGTAGCGGAGTGGGGTGTGCGTACGGAGTTCGGAGTGGACGCCAAGTCAGTCCTGCTG
ACTGAAACTGgGCATTCCGAAGCCTCCATTGACATTTCCGTTTCCGGTGAGAAGGACGTG
GAGGAGGGTGGTGACGTAGAGATGACGTGTCGTTGCCATGGCTGCACTTCCGCCGCGATA
TTCGACTGGTTCAAGGTGgCGTTTGCCGGAAGTGAGTGGGTGACAACCGGAAACTACACT
CATATCGCAGCCAAGGTCGACGTTGGCGTTTTGGGATTTCCAAACCCGATAGAGATTGAC
GATGGATTCGACCAGTTCAGCGTGACACCATCCAATTCCCTCCGTCTGACCGGGGCGCAG
GTAGCCGACGCAGGAAGGTACTGGTGTAAGGTGACGAGCGATGGGAGCGTGGACATCAAG
GCAACCGTGCTGAAGGTCAAAGTGCCGGAGTTCACCTGTGCCGGTAAGGCTGACGGGTAC
TACCCAGACCCGGAGGACTGCGCCATGTACTACCAGTGTCTGTACGGCTTCCCTCAGCCC
TTCCACCGCCCGTGTGGGTACGCCGGTATGGTCTTCAACCCCGAGCACCTGTACTGTGAC
TGGGCCTTCAACGTGGGACCGCCCTGTGGGAGCAAGGCTTAG
>VCBP1S2*01 [organism=Branchiostoma floridae] [molecule=DNA] [moltype=genomic] [location=genomic] [note=allele 01 of VCBP1 paralog 2; predicted transcript, Scaffold_82; Brafl1 genome resource animal] Amphioxus variable region-containing chitin-binding protein 1 (VCBP1), VCBP1S2*01, mRNA; VCBP1 allele (predicted)
ATGAAGTTGGTGCTTAGCTTGGTCGTTCTTGCTGTTGGCGCGCACGCCATGACCATCGTG
ACCGTCAGCACTCCTGAACCGAAGGTCGAGGCTAGTGAGCCTCCTACTATCGCCTGGTTC
AAGGGTAATGCCGATTTCCGCGGTGCCGAGCGGATCTACACGGGACACAAGGTGTGGGGG
AACGAGACGGAACGGCGGGAGGACAGCTTCGGGGACTACATCGGGCGGGTGGAGGTGGCG
GATCTGGACAAACCCGCCATCAAGATCAGCGGCATCAAGAATAATTGCAAAAACTTTTCC
CCTGCAGAGTACTTTGCGCGTTACTGGTGTACCGTAGCGGAGTGGGGTGTGCGTACGGAG
TTCGGAGTGGACGCCAAGTCAGTTCTGCTGACTGAAACTGGCCATTCCATGGTACATCCC
ATTTACATTTCCGTTTCCGGTGAGAAGGAAGTGGAGGAAGGTGGTGACGTAGAGATGACG
TGTCAGTGTCAAGGTTGCTTCTACCCAACATTCGAATGGTTCAAGGGCCCGGCGTTTGCC
GGAAGTGAGTGGGCGACGACCGGAAATTACACCCATATCGCATCCAAGGAATATTTTGGC
ATCATGGGATTTGTAGACCTAGAGATTGAGGATGGATTTGGCCAGTTCAGCGTGACGCCA

TCCAATTCCCTCCGTCTGACCGGGGCACAGGTGACCGACGCCGGAAGGTACTGGTGTAAG
GTGACGAGCGGTGGAATTGCGGACATCAAGGCCACCGTGCTGAAGGTCAAAGGCATGTAC
CCAAGGTCACTCACATTGCCGGAGTTCACCTGTGCCGGTAAGGCTGACGGGTACTACCCA
GACCCGGAGGACTGCGCCATGTACTACCAGTGTCTGTACGGCTTCCCCCAGCCCTTCCAC
CGCCCGTGTGGGTACGCCGGTATGGTCTTCAACCCGGAGCACCTGTACTGCGACTGGGCC
TTCAACGTGGGACCGCCATGTGGGAAGTGTGCCGGGCAAAAACTGAAGCGTGACAGCAAG
GACAAACTTAGAAAACGCTACCTGATTCGGCGACGCGTGGGGAGGGCGAGCTCAAGCTTG
AAGTAA
>VCBP1S3*01 [organism=Branchiostoma floridae] [molecule=DNA] [moltype=genomic]
[location=genomic] [note=allele 03 of VCBP1 paralog 3; predicted transcript, Scaffold_82; Brafl1 genome resource animal] Amphioxus variable region-containing chitin-binding protein 1 (VCBP1), VCBP1S3*01, mRNA; VCBP1 allele (predicted)
ATGAAGTTGGTACTTGGCTTGGTCCTTCTTAATCTTGCTGTTGGCGCGCACGCCATGACC
ATCGTGACCGTCAGTACCCCTGAACCGAAGGTCGAGGCTAGTGTTGGGGGTTCCGCGGAA
CTCAAGTGCGAGTTTGACATCCAACCCAACTCTACACAGCCTCCTACTATCGCCTGGTTC
AAGGGTAACGACGATTTCCGCGGTGCCGAGCGGATCTACACGGGACACAAGGTGTGGGGG
AACGAGACGGAACGGCGGGAGGACAGCTTCGGGGACTACATCGGGCGGGTGGAGGTGGCG
GATCTGGACAAACCCGCTATCAAGATCAGCGGCATCAGGAGTACTGACTTCGCGCGCTAC
TGGTGTACGGTAGCGGAGTGGGGTGTGCGTACGGAGTTTGGAGTGGACGCCAAGTCAGTT
CTTCTGACTGAAACTGGCAATTCCCTGGTACACCCTATTGACATTTCCGTTTCCGGTGAG
AAGGAAGTGGATGAAGGTGCTGATGTGCAGATGACGTGTCAGTGTCAAGGTTGCTTCTAC
CCTACATTCGACTGGTTCAAGGGCCCGGCCTTTGCCGGAAGTGAGTGGGTGACGACCGGA
AATTACACCCATATCGCATCCAAGGAATATCTCGGCATCATGGGATTTGTAGACCTAGAG
ATGGAGGACGGATTTGACCAGTTCAGCGTGACGCCTTCCAACTCCCTCCGTCTGACCGGG
GCGCAGGTAGCCGACGCAGGAAGGTACTGGTGTAAGGTGACGAACGGTGGGATTGCTGAC
ATCAAGGCAACCGTGCTGAAGGTCAAAGTTCCGGAGTTCACCTGTGCCGGTAAGGCTGAC
GGGTACTACCCAGACCCGGAGGACTGCGCCATGTACTACCAGTGTCTGTACGGCTTCCCT
CAGCCCTTCCACCGCCCGTGTGGGTACGCCGGCATGGTCTTCAACCCGGAGCACCTGTAC
TGCGACTGGGCCTTCAACGTGGGACCGCCATGTGGGCGCTTCAAGATGGCGGTGCGCGTT
AAGTGCAGTTACGGGCACTATTCCTTACTACGAATGACCATTGGGACAAAACCTGTTTTA
GCGAAGGAGCTGTTGGCTCACAGCGAAGGGCTACATGCTCGGCGAAGGAGCTGTGTGCTC
ACAGCAAAGGGGCTGCGTGCTCGCCGACGGGAGCTGTGTGCTCACAGCGAACTAGGCTGA
>VCBP4S1*03 [organism=Branchiostoma floridae] [molecule=DNA] [moltype=genomic]
[location=genomic] [note=allele 03 of VCBP4; predicted transcript, BAC5h9, Scaffold_295; note: lacks second exon of V2 domain; Brafl1 genome resource animal] Amphioxus variable region-containing chitin-binding protein 4 (VCBP4), VCBP4S1*03, mRNA; VCBP4 allele (predicted)
ATGGCGCCGTTCACACCGTTTGTCCTGTGTATCATCCTGGCAGGTGCTATGGCACAATCG
CCATTCCGAATCATGACAGTGACAGTGCCGAGGCCGGAAGTGACGGCAAACACCGGAAGT
GACATCAAACTGCCCTGTTCCTACGACATTCCGGCTGCCCGGTCCGGACTGAATCCGACC
ATCAATTGGTACAAGGGGGATGAAGGGATCGCCAGCGCTACCAAGATCTTCTCCCTCTCC
TACCACGGCTCGACACAAATAGCGGAGGCTTACGAGGGGTACGAGCTGCGGGTCGAACTG
GAGAGTCTCACCGACCCCACCCTGAAACTCAGCGGCGTCAGGCCTTCCGATCACGGTCGG
TACTGGTGCGCGGTGTTCGACTCCGACGAGCACAACCAATTCGGCATGGACTCCAAGTCG
GTTCTCCTCACAGTGATAGACCCTAGCGTGCAGCAGGACCAGGGTACGGGCCGCCCGGGC
CGTGTCGAGGTGCACGTGCCTGCTGTGCGGCAGGTGTACCAGGGGGAGGACGTGGAGCTC
CCCTGCGAGTGCGATGGGTGCCACTGGACCAGCATCAAGACTTGGTTCACGGTAGAAGAC
GGTACCTTCAACACTCGCGGCTCCGGAGTGGACGCTCAGTCCGTGGCTCTGGTCGTCGAG
TCACGTTGTGCGGGGAAGCCGGCCGGGCGCTACCAGCACCCTGACGACTGCTCCAAGTAC
TACACCTGCGGGGAGGGCGGGCTGCAGTACGACGGCATCAGTGCCTGTCCACCCGGCCTA
ATGTACGACCAGGCCAACGGCTACTGCAACTGGGCCACGCTCGTCACCTGTCTGTAA
>VCBP4S1*02 [organism=Branchiostoma floridae] [molecule=DNA] [moltype=genomic]
[location=genomic] [note=allele 02 of VCBP4; predicted transcript, BAC100j9 (EU875592), Scaffold_295; Brafl1 genome resource animal] Amphioxus variable region-containing chitin-binding protein 4 (VCBP4), VCBP4S1*02, mRNA; VCBP4 allele (predicted)
ATGGCGCCGTTTACCCCGTTTGTCCTGTGTACCATCCTGGCAAGTGCACTTGCACAATCG
CCATTCCGAATCATGACGGTGACCGTGCCGAGGCCGGAAGTGACGGCAAATGTCGGAAGT
GACGTCAAACTGCCCTGTTCCTACAGCATCCCGGCTGCCTCGTCCGGACTGAATCCGACC

ATCAACTGGTACAAGGGAGATGAAGGGATCGCCAGCGCTACCAAGATCTTCTCCCTCTCC
TACCACGGCTCGACACAAATAGCGGAAGCTTACGAGGGGTACGAGTTGCGGGTCGAACTG
GAGAGTCTCACTGATCCCACCCTGAAACTCAGCGGCGTCAGGGCGTCCGACCACGGCCGG
TACTGGTGCGCCGTGTTCGACTCCGACGAGCACAACCAATTCGGCATGGACTCCAAGTCG
GTTCTCCTCACAGTGATAGACCCTAGCATACAGCAGGACCAGGGAACTGGCCGCCCGGGC
CGTGTCGAGCTGCATGTGCCGGCTGTACGGCAGGTGTACCAGGGGGAGGACGTGGAGCTC
ACCTGCGAGTGCGATGATTGCCACTGGACTAGCACCAAGTCTTGGTTCACGGTGTCATTT
GATGACACTTGGGCGGCCACGGAGGAGCTGGTCGCTACCGTAGGTCCGTGCGTGAGCCAT
CAGTGCCATGGAGGGACCACCACCGTCGCCCCCGCATTCCGAGACCACTTCTCGGCAACT
CGCGAAGCCTTGAGGATTTCGGCAGCCAAGCGCCTGGACAAACGCAGGTACTGGTGTCAG
GTGGAAGATATGACCTTCAGTCGTGGCTCGGGAGTGGACGCTCAGTCCGTGGCTCTGGCT
GTCGAGTCACGTTGTGCGGGGAAGCCGGCCGGGCGCTACCAACACCCTGACGACTGCTCC
AAGTTCTACACCTGCGCGGAGGGCGGGCTGCAGCACGACGGCATCGGTGCCTGTCCGGAC
GGCCTGATGTACGATCAGGCACTCGGCTACTGCAACTGGGCCACGCTCGTCACCTGTCTG
TAA
>VCBP5S1*02 [organism=Branchiostoma floridae] [molecule=DNA] [moltype=genomic]
[location=genomic] [note=allele 02 of VCBP5; predicted transcript, BAC contig 63n5-43b24
(EU875590), Scaffold_82; Brafl1 genome resource animal] Amphioxus variable region-containing
chitin-binding protein 5 (VCBP5), VCBP5S1*02, mRNA; VCBP5 allele (predicted)
ATGTTGGGTCTACTGGTCAGCGTTCTTCTACTGCTTTATGTAGGACCGGAAAGGGTTGAT
GCGGTGTCCATCACAACCGTGACGGTGCCGGATCGAGGCGGTTGGGTGGTGATGTGGCCC
CGCCCAGGTGACCCTACCTGGGTGAACCGGATAGAGTTCAGGTGTGAATATATCATCTCA
CCTGCCTCGGCCACTCCTCCTACAATCACCTGGCTAAAGGGAGTGTTTGCCGACGCAGAC
CGACAGGTGGTCTACAAGTGGAGTTCATCAGGAGAAGTCTACGTCCATCCCGAGTTCGCA
GGCCGGGTCAGCGTGGAGTCCCGAACCCGCCCGACTCTCGTGCTGACCGACGCGAAGTTT
GACGACTGGGGCCGATACTGGTGTCGGGTTACGAACGAGGACCAGTCGGACGAGTTCGGC
ACGAACGAGGAGTCTCTGCTCTTCTGGTACAAGTTGGGCTACGACCCACCGAACCGTCTG
TCGCAGGTTAGCCTTGACAAAACCCCAGTCCGCGTGGCTGCTGGCGAGACGGCTCGGCTC
GACTGTACGGGAACCAGCGGCCGTGAAGCCTCCATTCTCTGGGTCAAAGGGCCGACGGGT
TGTACCCAAGGTGGAAGCTGTGACACTTACGAGACTGTCATTCACAAGTCCGCCGTGTGG
GGCGCTGGTAATCCAGAACCGGAAAACATCACCATCTCCCCCAGCTTCAACGGAAGGGTT
TCCCTGGATGCCGACGGGTCTGGCTCCTTCACTCCGACCCTGACCATCACCGACATCCGC
CCTAGCGACTCCGGCCGGTACTGGTGCGCTCCTGACATCTCCGAGGATTACTCTAACCTG
GGTCCTCTGAACCGGGACGCGCAGTCTGTGGTCATCATCGTCAACGACCCAGGTACCGAG
CCGACCTGTGCCGGTAAGCCGGACGGGATGTACCAGCACCCCGCCGACTGTGCCCAGTTC
TACACGTGCTCGGGCGGTCTGTCTTACGGCACCAACACCTGCCCAGCTGGACTGGTCTTC
AACCAGGAACTACAGCTGTGCGACTGGGCAAACAACGTCATCTGTGTGTAA
>VCBP5S2*01 [organism=Branchiostoma floridae] [molecule=DNA] [moltype=genomic]
[location=genomic] [note=allele 01 of VCBP5 paralog 2; predicted transcript, BAC contig
63n5-43b24 (EU875590), Scaffold_82; Brafl1 genome resource animal] Amphioxus variable region-
containing chitin-binding protein 5 (VCBP5), VCBP5S2*01, mRNA; VCBP5 allele (predicted)
ATGCTGGGTCTGTTGGTCGCTATCTCCGCTGTAGCGTGCTTTGAATCAAGCTACGCAGAT
GCAGTGTCCATCACGACCGTGACGGTACCGTATCACAATTATTACGTGTTGGCGGCGGAC
CGCCCGTGGGACCCTACCTGGGTGAACAGGGTAGAGATTAGGTGTGAATATACCATCTCA
CCTGCACCAGCCACTCCTCCTACTATCACCTGGCTCAGGGGAACCTTTGCAGACCGAGAG
GTGGTGTACAAGTGGAGCTCTTCAGGAGAAGTGTACGTGCATCCCGAGTTCGCAGGCCGC
GTCAGTGTGGAGTCCAGGACCCGGCCTACGCTGGTATTGTTCAACGAAAGGACCTGGGTT
GACCGATACTGGTGCCGGGTCACGAACGAGGAGCAACCGGACGAGTTCGGCACGGACGAG
GAGTCTTGCGGCTTCTGGTACGTTGGGATTGGCTATAACTTGCCTAAACTTCTGGCGACA
GTTCACCTGGACAAAACCCCGGTCCATGTGGATGCTGGCGGGACAGTCCAACTCAACTGT
ACGGGAGTCCCAGACGGCCGTATGGCTACCATTTTCTGGGTCAAAGGGCCGAGCTGCACT
CAAGGTGGAAGTTGTGACAGTTATGAGACTGTGATTCACAAGTCCTCCGGGTGGGGCGAA
ACGGAAACCATTACCGTCTCCCCCAACTTCGCTGGAAGGGTTTCTCTGGCTACCAGGGGT
GTGTCTATCACCCCGACCCTGACCATCACCGACATTCGCCCTAGAGACGCCGGTCGGTAC
TGGTGCTCTATAAACTACCCCGATGATCGCTACTCTTCTAGTTTTGGCCGTTGGAACCGG
GGCGCTCAGTCTGTGGTCATCCTCGTCAACGACCCAGTTACCGAGCCGACCTGTGCCGAT
AAGGCGGACGGGAAATACCAGCACCCCGCCGACTGTGCCCAGTTCTACACGTGCTCGGGC

GGTCTGTCTTACGGCACCAACACCTGCCCGGCTGGACTGGTCTTCAACCAGGACCTACAG
CTGTGCGACTGGGCAAACAACGTCATCTGCCTGTAG
>VCBP2S1*02 [organism=Branchiostoma floridae] [molecule=DNA] [moltype=genomic] [location=genomic] [note=allele 02 of VCBP2; predicted transcript, BAC contig 63n5-43b24 (EU875590), Scaffold_82; Brafl1 genome resource animal] Amphioxus variable region-containing chitin-binding protein 2 (VCBP2), VCBP2S1*02, mRNA; VCBP2 allele (predicted)
ATGTTGGGTCTGTTGGTCGCTGTCTCCGCCGTAGCGTGCTACGCAGGTGCGGTGTCCATC
ACGAACGTGACGGTACCGGATCGAAGCGCCGCGGCCGGTTGGGTCTATCTTCGATACAGC
CCGGACCCTACCTGGGTGAACAGGATAGAGTTCAGGTGTGAATATACCATCTCACCTGCC
TCAGCCAATCCGCCTACTATCACCTGGCTCAAGGGACCCTTCACAGACCGACAAGTGATC
TACAAGTGGAGTTCATCAGGAGAAGTCTACGTCCATCCCGAGTTCGCAGGCCGGGTCAGC
GTGGAGTCCCGAACCCGCCCGACTCTCGTGCTGACCGACGCGAAGTATGACGACTGGGGC
CGATACTGGTGTCGGGTCACGAACGAGGACCAATCGGACGAGTTCGGCACTGACGAGGAG
TCTCTGCTATTCTATTACAAGTCGACTGTCTACGATTACGACGTCCCTGCTCGTGGCGGC
CAATATTCTTTTGTGGAGGTGGACAAAACACCAGTCCGTGTGAAGGCGGGTGGGACGGCC
CGGCTCAACTGTGAGGGATCGGGCGGCCCTCTGGCGTCCATTGTCTGGTTCAAAGGGCCA
AGTTGTACCCAAGACGGAAAGTGCAACGTATATGAGATGGTCATCAACAAAACAGCTGTA
CCTCAGCCCCATCCGATTCTAGGTCCGGGAACCGTTAACGTGTCGCCGAAGTACGCAGGA
AGGGCTTCACTGGACTTCAACGACGGTGGCTACTACAATTATTACTATCCGGATCTAACC
ATCACTGACATTCGCCCTACCGACGTCGGTCGGTACTGGTGCACCGATGATGCGCCCTTA
TGGTACCAGAATGATCTGCGCAGCCGCGATTCCCAGTCTGTGGTGGTGCTCCTTGACGAT
GAAGCACCAACACCGTCATGTGATGGGAAGGCTGACGGGATGTACCAGGACCCTGGCGAC
TGTTCCCGGTACTACAGCTGCTCGGGTGGCTGGCTGTACGGCCCAGCGCCCTGTCTGACC
GGGCTGTTCTTTAACGAGGCCTTGCAAGTGTGTGACTGGCCTAATAACGTTGCCTGTGCG
TAA
>VCBP5S2*02 [organism=Branchiostoma floridae] [molecule=DNA] [moltype=genomic] [location=genomic] [note=allele 02 of VCBP5 paralog 2; predicted transcript, BAC62d19 (EU875589), Scaffold_295; Brafl1 genome resource animal] Amphioxus variable region-containing chitin-binding protein 5 (VCBP5), VCBP5S2*02, mRNA; VCBP5 allele (predicted)
ATGCTGGGTCTGTTGTTTGCTATCTCAGCTGTAGCTTGCTTTGAATCAAGCTACGCAGAT
GCGGTGTCAATCACGACTGTGACGGCACAGAACGGTGTTGTCTTCGCGTATAACCGCTGG
TGGGACCCTACCTGGGTAAACAGGGTAGAGTTCAGGTGTGAATATACCATCTCACCTGCA
CCAGCCACTCCTCCTACAATCACCTGGCTCAGGGGAGTGTTTGCAGACCAAGAAGTGATC
TACAAGTGGAGTTCATCAGGAGAAGTCTACGTGCATCCCGAGTACACAGGCCGCGTCAGT
GTAGAGTCCAGGACCCGGCCTACGCTGGTATTGTTCAACGAAACGGTGTGGGCCAACCGA
TTCTGGTGCCGGGTCACCAACGAGGAACAACCGGGCGAGTTCGGCATGGACGAGGAATCT
AGCATCTTCTGGTTTCAAACTGGCAGCGACTGGCCTAAAAGTCTGTCGCATGTTAGCCTG
GACAAAACCCCGGTCCATGCGGATGCTGGAGGGACAGTCCAACTCAACTGTACGGGAGGC
CCAACCGGCCGTTTGGCTACCATTTTCTGGGTCAAAGGGCCGAGCTGCACTCAAGGTGGA
AGTTGTGACAGTTATGAGACTGTGATTCACAAGTCCTCCGGGTATGGCGAAACGGAAACC
GTCACCGTCTCCCCCAACTTTGCTGGAAGGGTTTCCCTGGCTACCAGGCATCCCTTCTCC
GACATCACCCCGACCCTGACCATCACCAACATCCGCCCTAGCGACGCCGGCCGGTACTGG
TGCTCAACTAACTACGCCGATATCTACTCTTCTAGTTTGGGCCCTTGGAACCGGGGCGCC
CAGTCTGTGGTCATCCTCGTCAACGACCCAGGTACCGAGCCGACCTGTGCCGGTAAGCCA
GACGGGATGTACCAGCACCCCGCCGACTGTGCCCAGTTCTACACGTGCTCGGGCGGTCTG
TCTTACGGCACCAACAACTGCCCGGCTGGACTGGTCTTCAACCAGGAACTGCAGCTGTGT
GACTGGGCAAACAACGTCATCTGTCTGTAG
>VCBP2S1*03 [organism=Branchiostoma floridae] [molecule=DNA] [moltype=genomic] [location=genomic] [note=allele 03 of VCBP2; predicted transcript, BAC62d19 (EU875589), Scaffold_295; Brafl1 genome resource animal] Amphioxus variable region-containing chitin-binding protein 2 (VCBP2), VCBP2S1*03, mRNA; VCBP2 allele (predicted)
ATGTTGGGTCTGTTGGTTGCCATCTCCGCTGTAGCGTGCTACGCAGGTGCTTTGTCCATC
ACCAACGTGACGCTACCGGATCGAAGCGCCGGTTGGGTCTATCTTCGGTACAGCCCGGAC
CCTACCTGGGTGAACAGGATAGAGTTCAGATGTGAATATTCCATCTCACCTGCACCAGCC
ACTCCTCCTACTATCACCTGGCTCAAGGGACCCTTCACAGACGCAGACCGACTGGTGGTC
TACAAGTGGAGTTCATCAGGAGAAGTCTACGTCCATCCCGAGTTTGCAGGACGGGTCAGC
GTACCGTCCCGAACCCGCCCGACTCTCGTGCTGACCGACGCGAAGTTTGACGACTGGGGC

CGATACTGGTGTCGGGTCACAAACGAGGAACAATCGGACGAGTTCGGCACGGACGAGGAG
TCTCTGCTCTTCTGGTACAAGTCCACTGTCTACGATTACGACGCCCCTCCCCGTGGCGGC
TATTCTTCTTTTGTGGAGGTGGACAAAACACCAGTCCGTGTGAAGACCGGTGGGACAGCC
CGGCTAAACTGTCAGGGAGCCGGCGGCCCTCTGGCGTCCATTGTTTGGTTCAAAGGGCCA
AGCTGTTCCCAAGACGGAAATTGCAACGTATATGAGATCGTCATCAACAAAACTGCTGTA
CCTCAGCCTCATGCGATTCTAGGTCCGGGAACCGTTAACGTGTCGCCGAAGTACGCAGGA
AGAGCTTCACTGGACTTCAACGACGGACCCTACTACAATGTTTACTATCCGGATCTAACC
ATCACTGACATTCGCCCTTCCGACGTCGGCCGGTACTGGTGCACCAATGATGCACCCTTA
TGGTACCAGAATGATCTGCGCAGCCGCGATTCCCAGTCTGTGGTGGTGCTCCTTGACGAT
GAAGCACCGACACCGTCATGTGATGGGAAAGCGGACGGGATGTACCAGGACCCTGGCGAC
TGTTCCCGGTACTACACCTGCTCGGGTAGCTGGCTGTACGGCCCAGTGCCCTGTCTGACA
GGGCTGTTCTTTAACGAAGCCCTGCAAGTGTGTGACTGGCCTAATAACGTTGCCTGTGCG
TAA
>VCBP5S1*03 [organism=Branchiostoma floridae] [molecule=DNA] [moltype=genomic]
[location=genomic] [note=allele 03 of VCBP5; predicted transcript, BAC62d19 (EU875589),
Scaffold_295; Brafl1 genome resource animal] Amphioxus variable region-containing chitin-binding
protein 5 (VCBP5), VCBP5S1*03, mRNA; VCBP5 allele (predicted)
ATGTCGATTCTACTGGTTATCATTATTCTACTGCTTTACGTAGGACCGGAAAGTGCGGAT
GCGGTGTCTATCACGACCGTGACGGTACCGGATCGAGGCGGTTGGGTGGTGATGTGGCCC
CGCCTGGGTGACCCTACCTGGGTGAACCGGATAGAGTTCAGGTGTGAATATTCCATCTCA
CCTGCCTCAGCCACTCCGCCTACTATCACCTGGCTAAAGGGGGTGTTTGCCGACGAAGAC
CGACAGGTGATCTACAAGTGGAGTTCATCAGGAGAAGTCTACGTCCATCCTGAGTTCGCA
GGACGGGTCAGCGTACCATCCAGAACCCACCCGACTCTCGTGCTGACCGACGCGAAGTTT
GACGACTGGGGCCGATACTGGTGTCGGGTCACGAACGAGGACCAATCGGACGAGTTCGGC
ACGGACGAAGAGTCTCTGCTCTTCTGGTACAAGTTGGGCTACGACCCACCGACCCGTCTG
TCGCAGGTTAACCTGGACAAAACGCCAGTCCGCGTGGCTGCTGGAGGGACAGCTCGGCTC
GACTGTACGGGAACCAGCGGCCGTGAAGCCTCTATTCTCTGGGTCAAGGGGCCGGCGGAC
TGTACTCAAGGTGGAAGCTGTGACAGTTATGAGACTGTCATTCACAAGTCCGCCGTGTGG
GGCGCTGGTAATCCAGAACCGGAAAACATCACCATCTCCCCCAGCTTCGACGGAAGGGTT
TCCCTGGATGCCGACGGGTCTGGCTCCTTCACCCCGACCCTGACCATCACCGACATCCGC
CCTAGCGACTCCGGCCGGTACTGGTGCGCCCCTGACATTTCCGAGGATTACTCTAACCTG
GGTCCTCTGAACCGGGACGCGCAGTCTGTGGTCATCATCGTCAACGGCCCAGGTACCGAG
CCGACCTGTGCCGGTAAGCCGGACGGGATGTACCAGCACCCCGCCGACTGTGCCCAGTTC
TACACGTGCTCGGGCGGTCTGTCTTACGGCACCAACACCTGCCCAGCTGGACTGGTCTTC
AACCAGGAACTACAGCTGTGCGACTGGGCAAACAACGTCATCTGTGTGTAA
>VCBP3S1*02 [organism=Branchiostoma floridae] [molecule=DNA] [moltype=genomic]
[location=genomic] [note=allele 02 of VCBP3; predicted transcript, Scaffold_1, BAC90f15
(EU875591); Brafl1 genome resource animal] Amphioxus variable region-containing chitin-binding
protein 3 (VCBP3), VCBP3S1*02, mRNA; VCBP3 allele (predicted)
ATGCAGGTGTTTCTACTCGTCTCCATATGCCTCGGCATGGCCTATGGGCAGTCCATCATG
ACCGTCCGCACCACCCACACAGAAGTAGAGGTCCATGCCGGCGGTACCGTGGAGCTCCCC
TGTGCGTACCAGCTGGCGAACGACACCCAGCCTCCCGTCATCTCATGGCTCAAGGGGGCA
TCACCGGACAGGAGCACCAAGGTCTTCAAGGGGAACTACAACTGGCAGGGAGAAGGGCTG
GGGTTCGTGGAGAGCGACAGCTACAAGGAGAGTTTCGGGGACTTCCTCGGTCGGGCCTCG
GTGGCAAACCTGGCCGCGCCCACTCTGCGGCTTACTCACGTTCACCCACAGGACGGTGGG
CGGTACTGGTGTCAGGTGGCGCAGTGGAGTATCCGGACAGAGTTCGGGCTGGACGCCAAG
TCCGTGGTGCTGAAGGTGACAGGCCACACCCCATCCAATAATGTCCACGTCTCCACGGCG
GAAGTTGTCCAGGTTGACGAGGGCAATGACATTACCATGACGTGCCCCTGCACCGACTGC
GCAAACGCCAACGTCACGTGGTACACCGGGCCAACCTTCTTCGAGAACTACGAGACCGGC
ACCTACCAACCACTGGCTAACAAGAACCAGTTCGGCATCACCTGGTTCTCGTCTGAGATC
GCGGGCCGGGCGAGCTTCAGCGGCGCGCGCAACCTGGTTCTTCGGGCCGCCAAGATCACA
GACGCCGGCCGCGTCTGGTGCGAGCTCGCGACTGGACAGGGAGAGCTAGACGCGGACAGG
TCCTCAACCATCCTCAAGGTCCAACTGGAACCGTTCACCTGTGACGGTAAGCCGACTGGC
CTGTACGCCGACCCCACCGCCTGTGACTACTACTACCAGTGCATCCCTGGCTATCCTCCC
CTACACCGCCCCTGCGGGTATGCCGGCATGGTATTCAACGAGGAGATGCAGTACTGCGAC
TGGGACATCAACGTGCCGCCACCTTGCGGAAGCAAGCCGGTGTGA
>VCBP3S1*03 [organism=Branchiostoma floridae] [molecule=DNA] [moltype=genomic]

[location=genomic] [note=allele 03 of VCBP3; predicted transcript, BAC54h3 (EU875588), allele of Scaffold_1; second exon excluded; Brafl1 genome resource animal] Amphioxus variable region-containing chitin-binding protein 3 (VCBP3), VCBP3S1*03, mRNA; VCBP3 allele (predicted)
ATGACCGTCCGCACGACCTATACAAAAGTGGAGGTGCACGCAGGCGGTACCGGGAACTAC
AACTGGCAGGGTGAAGGGTTGGGGTTCGTGGAGAGCGACAGCTACAAGGAGAGTTTCGGG
GACTTCCGCGGACGGGCCTCTGTGGCAAACCTGGCCGCGCCCACTCTGCGGCTCACTCAC
GTCCATCCACAGGACGGTGGCCGGTACTGGTGCCCGGTGGCGCAGTGGAGCATCCGGACC
GATATTACCCCTTCAAACCCACCAGGTCATACGCCATCCAACAACGTTCACGTCTCCACG
GCGGAAGTTGTCCAGGTTGACGAGGGCAATGACATTACCATGACGTGCCCCTGCACTGAC
TGCGCAAACGCCAACGTCACGTGGTACACCGGGCCAACCTTCTTCGAGAACTACGAGACC
GGCACCTACCAGCCTCTAGCCAACAAGAACCAGTTCGGCATCACCTGGTTCTCGTCTGAG
ATCGCGGGCCGTGCGAGCTTCAGCGGTGCGCGCAACCTGGTCCTTCGGGCCGCCAAGATC
ACAGACGCCGGCCGCGTCTGGTGCGAGCTCGCGACCGGCCAAGGCGAGCTGGATGCAGAC
AGGTCCTCAACCATTCTCAAGGTCCAACTGGAGCCGTTCACCTGTGATGGGAAGCCGACG
GGCCTGTACGCCGACCCCACCGCCTGTGACTACTACTACCAGTGTATCCCTGGCTATCCT
CCCCTCCACCGCCCCTGCGGCTACGCCGGCATGGTGTTCAACGAGGAGATGCAGTACTGC
GACTGGGACTTCAACGTGCAATCACCTTGCGGAAGCAAGCCGGTGTGA
>VCBP3S1*04 [organism=Branchiostoma floridae] [molecule=DNA] [moltype=genomic]
[location=genomic] [note=allele 04 of VCBP3; predicted transcript, PAC30b18 (AC135602.14), independent to genomic resource, Brafl1] Amphioxus variable region-containing chitin-binding protein 3 (VCBP3), VCBP3S1*04, mRNA; VCBP3 allele (predicted)
ATGCAGCCGTTTCTGCTTGTCTCCCTGTGCATTGGCATGGCCTATGGCCAGTCCATCATG
ACAGTCCGCACGACCCATACAGAAGTAGAAGTGCACGCCGGCGGTACCGTGGAGCTCCCC
TGCGCCTACCAGCTTGCTAACGACACCCAGCCTCCCGTCATCTCATGGCTCAAGGGGGCC
TCACCGGACAGGAGCACCAAGGTCTTCAAGGGAAACTACAACTGGCAGGGAGAAGGGCTG
GGGTTCGTGGAGAGCGACAGCTACAAGGAGAGTTTCGGGGACTTCCGCGGACGGGCCTCG
GTGGCAAACCTGGCCGCGCCAACTCTGCGGCTTACTCACGTCCACCCACAGGACGGTGGC
CGGTACTGGTGCCAGGTGGCGCAGTGGAGCATCCGGACAGAGTTTGGGCTGGATGCCAAG
TCCGTGGTGCTCAAGGTGACAGGCCACACCCCATCCAATAATGTCCACGTCTCCACGGCG
GAAGTTGTCCAGGTTGACGAGGGCAATGACATTACCATGACGTGCCCCTGCACTGACTGC
GCAAACGCCAACGTCACGTGGTACACCGGGCCAACCTTCTTCGAGAACTACGAGACCGGC
AGCTACCAGCCTCTGGCCAACAAGAACCAGTTCGGCATCACCTGGTTCTCGTCTGAGGTC
GCGGGCCGGGCGAGCTTCAGCGGTGCGCGCAACCTGGTCCTCCGGGCCGCCAAGATCACT
GACGCCGGCCGCGTCTGGTGCGAGCTCGCGACCGGCCAGGGCGAGCTGGATGCAGACAGG
TCCTCAACCATTCTGAAGGTCCAACTGGAGCCGTTCACCTGTGACGGGAAGCCGACAGGC
CTGTATGCCGACCCCACCGCCTGTGACTACTACTACCAGTGTATCCCCGGTTACCCTCCC
CTCCACCGCCCCTGCGGGTATGCCGGCATGGTCTTCAACGAGGAGATGCAGTACTGCGAC
TGGGACATCAACGTGCCGCCACCTTGCGGAAGCAAGCCGGTGTGA
>VCBP4S1*04 [organism=Branchiostoma floridae] [molecule=DNA] [moltype=genomic]
[location=genomic] [note=allele 04 of VCBP4; predicted transcript, PAC34i17 (AC141434.12), independent to genomic resource, Brafl1] Amphioxus variable region-containing chitin-binding protein 4 (VCBP4), VCBP4S1*04, mRNA; VCBP4 allele (predicted)
ATGGCTCCGTTCACACCGTTTGTCCTGTGTATCATCCTGGCAGGTGCTATGGCACAATCG
CCATTCCGAATCATGACGGTGACCGTGCCGAGGCCGGAAGTGACGGCAAATGTCGGAAGT
GACGTCAAACTGCCCTGTTTCTACAGCATCCCGGCTGCCTCGTCCGGACTGAATCCGACC
ATCAACTGGTACAAGGGAGATGAAGGGATCGCCAGCGCTACCAAGATCTTTTCCCTCTCC
TACCACGGCTCCACACAAATAGCGGAGGCTTACGAGGGGTACGAGTTGCGGGTCGAACTG
GAGAGTCTCACTGATCCCACCCTGAAACTCAGCGGCGTCAGGGCGTCCGACCACGGCCGG
TACTGGTGCGCCGTGTTCGACTCCGACGAGCACAACCAATTCGGCATGGACTCCAAGTCG
GTTCTCCTCACAGTGATAGACCCTAGCATACAGCAGGACCAGGGTATGGGCCGCCCGGGC
CGTGTCGAGCTGCGTGTGCCGGCTGTACGGCAGGTGTACCAGGGGGAGGACGTGGAGCTC
ACATGCGAGTGCGATGATTGCCACTGGACTAGCACCAAGTCTTGGTTCACGGTGTCATTT
GATGACACCTGGGCGGCCACGGAGGAGCTGGTCGCTACCGTAGGTCCGTGCGTGAGCCAT
CAGTGCCATGGAGGGACCACCACCGTCGCCCCCGCATTCCGAGACCACTTCTCGGCAACT
CGCGAAGCCTTGAGGATTTCGGCAGCCAAGCGCCTGGACAAACGCAGGTACTGGTGTCAG
GTGGAAGATATGACCTTCAGTCGTGGCTCGGGAGTGGACGCTCAGTCCGTGGCTCTGGCT
GTCGAGTCACGTTGTGAGGGGAAGCCGGCGGGGCGCTATCAACACCCTGACGACTGCTCC

AAGTACTACACCTGCGCGGAAGGCGGGCTGCAGTACGACGGCATCAGTGCCTGTCCACCC
GGCCTAATGTACGACCAGGCCAACGGCTACTGCAACTGGGCCACGCAGGTCACCTGTCTG
TAA
>VCBP2S1*04 [organism=Branchiostoma floridae] [molecule=DNA] [moltype=genomic] [location=genomic] [note=allele 04 of VCBP2; predicted transcript, PAC37d15 (AC135603.5), independent to genomic resource, Brafl1] Amphioxus variable region-containing chitin-binding protein 2 (VCBP2), VCBP2S1*04, mRNA; VCBP2 allele (predicted)
ATGTTGGGTCTGTTGGTCGCTATCTCCGCCGTAGCGTGCTACACTGGTGCGGTGTCCATC
ACGAACGTGACGGTACCGGATCGAAGCGCCGCGGCCGGTTGGGTCTATCTTCGATACAGC
CCGGACCCTACCTGGGTGAACAGGATAGAGTTCAGGTGTGAATATACCATCTCACCTGCC
TCAGCCAATCCGCCTACTATCACCTGGCTCAAGGGACCCTTCACAGACCGACAAGTGATC
TACAAGTGGAGTTCATCAGGAGAAGTCTACGTCCATCCCGAGTTCGCAGGCCGAGTCAGT
GTACCATCCCGAACCCGCCCGACTCTCGTGCTGACCGACGCGAAGTTTGACGACTGGGGC
CGATACTGGTGTCGGGTCACGAACGAGGACCAATCGGACGAGTTCGGCACTGACGAGGAG
TCTCTGCTATTCTATTACAAGTCGACTGTATTCATGACCAAGAATGCCACACTCACACTT
GTTTGTGCAGTCTACGATTACGACGTCCCTGCTCGTGGCGGCCAATATTCTTTTGTGGAG
GTGGACAAAACGCCAGTCCGTGTGGAGACAGGTGGGACGGCCCGGCTCCACTGTGAGGGA
TGGGGCGGCCCTCTGGCGTCCATTGTCTGGTTCAAAGGGCCAAGTTGTACCCAAGACGGA
AATTGCAACGTATATGAGATGGTCATCAACAAAACAGCTGTACCTCAGCCCCATCCGATT
CTAGGTCCGGGAACCGTTAACGTGTCGCCGAAGTACGCAGGAAGGGCTTCACTGGACTTC
AACGACGGTGGCTACTACAATTATTACTATCCGGATCTAACCATCACTGACATTCGCCCT
ACTGACGTCGGTCGGTACTGGTGCACCGATGATGCGCCCTTATGGTACCAGAATGATCTG
CGCAGCCGCGATTCCCAGTTTGTGGTGGTGCTCCTTGACGATGAAGCACCAACACCGTCA
TGTGATGGGAAGGCTGACGGGATGTACCAGGACCCTGGCGACTGTTCCCGGTACTACACC
TGCTCGGGTGGCTGGCTGTACGGCCCAGTCTCCTGTCTGACAGGGCTGTTCTTCAACGAG
GCCTTGCAAGTGTGTGACTGGCCTGATAACGTTGCCTGTGCTTAA
>VCBP5S1*04 [organism=Branchiostoma floridae] [molecule=DNA] [moltype=genomic] [location=genomic] [note=allele 04 of VCBP5; predicted transcript, PAC37d15 (AC135603.5), independent to genomic resource, Brafl1] Amphioxus variable region-containing chitin-binding protein 5 (VCBP5), VCBP5S1*04, mRNA; VCBP5 allele (predicted)
ATGTCGGTTCTACTGGTCAGCATTATTCTACTGCTTTACGTAGGACCGGAAAGTGCAGAT
GCGGTGTCTATCACGACCGTGACAGTACCAGATCGAGGCGGTTGGGTGGTGATGTGGCCC
CGCCCAGGTGACCCTACCTGGGTGAACAGGATAGAGTTCAGGTGTGAATATTCCATCTCA
CCTGCTTCAGCCACTCCTCCTACTATCACCTGGCTCAAGGGAGTGTTTGCCGACGCAGAC
CGACAGGTGATCTACAAGTGGAGTTCATCAGGAGAAGTCTACGTCCATCCCGAGTACGCA
GGCCGGGTCAGTGTACCGTCCCGAACCCACCCGACTCTCGTGCTGACGGACGCGAAGTTT
GACGACTGGGGCCGATACTGGTGTCGGGTCACGAACGAGGACCAATCGGACGAGTTCGGC
ACGGACGAGGAGTCTCTTCTCTTCTGGTACAAGTTGGGCTACGACCCACCGACCCGTCTG
TCGCAGGTTAGCCTTGACAAAACGCCAGTCCGCGTGGCTACTGGTGGGACGGCTCGGCTG
GACTGTACGGGAACCAGCGGCCGTGAGGCATCCATTCTCTGGGTCAAAGGGCCGACAAGC
TGTACTCAAGGTGAAAGCTGTGACAGTTACGAGACTGTCATTCACAAGTCCGCCGTGTGG
GGCGCTGGTAACCCAGAACCGGAAAACATCACCATCTCCCCCAGCTTCGACGGAAGGGTT
TCCCTGGATGCTGACGGGTCTGGCTCCTTCACTCCGACCCTTACCATCACCGACATCCGC
CCTAGCGACTCCGGCCGGTACTGGTGCGCTCCTGACATCTCCGAGGTTTACTCTAACCTG
GGTCTGCTGAACCGGGACGCTCAGTCTGTGGTCATCATCGTCAACGACCCAGGTACAGAG
CCGACCTGTGCCGGTAAGCCGGACGGGATGTACCAGCACCCCGCCGACTGTGCCCAGTTC
TACACCTGCTCGGGCGGTCTGTCTTACGGCACCAACAACTGCCCGGCTGGACTGGTCTTC
AACCAGGAACTCCAGCTGTGCGACTGGGCAAACAACGTCATCTGTCTGTAG
>VCBP5S2*03 [organism=Branchiostoma floridae] [molecule=DNA] [moltype=genomic] [location=genomic] [note=allele 03 of VCBP5 paralog 2; predicted transcript, PAC37d15 (AC135603.5), independent to genomic resource, Brafl1] Amphioxus variable region-containing chitin-binding protein 5 (VCBP5), VCBP5S2*03, mRNA; VCBP5 allele (predicted)
ATGTTGGGTCTGTTGGTCGCCATCTCCGCTGTAGCGTGTTTTGAATCAAGTTACGCAGAC
GCGGTGTCCATCACGACCGTGACGGTACCGAATCGAGGCGGTTGGGTGTTGGTGTGGCCC
CGCCCAGGTGACCCTAACTGGGTGAACCGGGCAGAGATCGGGTGTGAATATACCATCTCA
TCTGCACCAGCCACTCCTCCTACTATCACCTGGCTCAGGGGAACATTCGCCGACAAACAG
GTGATCTACAAGTGGAGCTCTTCAGGAGAAGTCTACGTGCATCCCGACTTCGCAGGCCGG

GTCAGTGTGGAGTCCAGGACACATCCTACACTGATACTGGTCAACCAAGGAGGCTGGGGC
CGATACTGGTGTCGGGTCACCAACGAGGAGCAACCCGGCGAGTTCGGTACAGACGAGGAG
TCTCGCCTCTTTTGGTACGGTTCAGGATACGACTTGCCTGGCCGTCTGTCCTGGCTTAAC
CTGGACAAGACCCCGGTCCATGTGGAGGTTGGCGGGACAGTCCGACTCAACTGTAACGGA
ACCAGTGGCTCTACCGCTTCCATTCTCTGGGTCAAAGGGCCGAGCTGCATTCAAGGTGGA
GGCTGTGACAGTTACGAGACTGTAATTCACAAGTCTGCCCAGTACGGCACTTCCAACACA
GAACCGGAAATCACCATCTCCCCCAACTACACTGGAAGGGTTTCCTTGGATACACAATAT
TACACCGTAAATGACTACACCTACTTGGAATTCACCCCCACCCTGACCATCACTGACATC
CGCCCTAGCGACGCCGGTCGGTACTGGTGCTCTCCTGCCCACACGGAATTGTACTCTTAT
CTGGGCACTCTGAACCGGGACGCTCAGTCTGTGGTCGTCATCGTCAACGACCCAGGTACC
GAGCCGACCTGTGCGGGTAAGCCGGACGGGATGTACCAGCACCCCGCCGACTGTGCCCAG
TTCTACACGTGCTCGGGCGGTCTGTCTTACGGCACCAACACCTGCCCAGCTGGACTGGTC
TTCAACCAGGAACTCCAGCTGTGCGACTGGGCAAATAACGTGATCTGTCTGTAG
