# Supplementary material for: Genomic complexity of the variable region-containing chitin-binding proteins in amphioxus
Source: BMC Genet. 2008 Dec 1;9:78. doi: 10.1186/1471-2156-9-78 (PMC2632668; doi:10.1186/1471-2156-9-78)
Supplement: Additional file 2 — Dot plot pairwise comparison of the VCBP2/5 cluster containing BAC clones, 62d19 and 63n5-43b24 contig (haplotype B and A, respectively), reveals extensive polymorphism. [file 1471-2156-9-78-S2.pdf]

Additional file 2: Dot plot pairwise comparison (window size of 11) of the VCBP2/5 cluster containing BAC clones, 62d19 and 63n5-43b24 contig (haplotype B and A, respectively), reveals extensive polymorphism. Arrow highlights the region encoding the VCBP2/5 cluster. Three large prominent repeats [(a) – (c)] across the two allelic regions (see Additional file 3) flank the VCBP2/5 cluster. Repeat (a) is ~6 kb in 62d19, ~3 kb in the paired allele, predicted to be non-coding and lacks significant homology to other sequences in the databases. Length variants of (a) are ubiquitous in the Bf genome and include short segments (~11-14 nucleotides; see Additional file 3). Repeat (b) is ~54 kb on contig 63n5-43b24, contains interspersed copies of repeats from (a) as well as other alternating short simple sequence elements, contains fragmented copies of an ancient gypsy-type mobile element and exhibits interallelic length variation (see Additional file 3). Repeat (c) is non-coding and lacks significant homology to other known sequences; elements of (c) are found throughout the locus [Additional file 3, as seen in (a)]. All three repeats contain alternating units of simple sequences, some of which form palindromic pairs with complementary partners distributed within the locus. A highly conserved VCBP5-type CBD pseudogene (single exon) is localized downstream of repeat (b) in BAC contig 63n5-43b24 (haplotype A) and is present on the opposite allele in the reference genome (arrowhead).

Figure S1

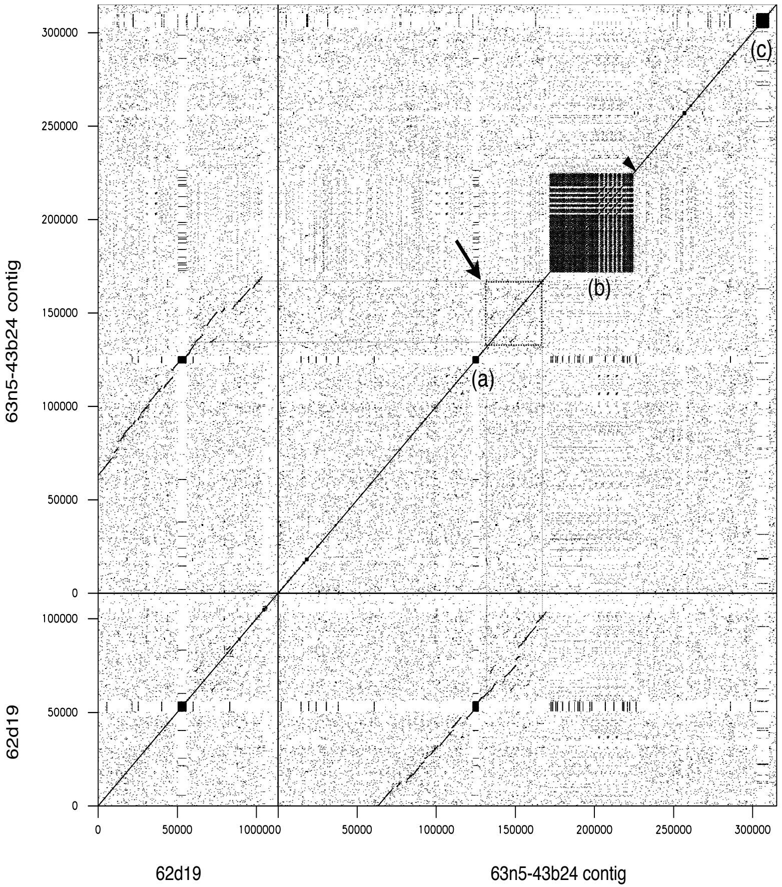

63n5-43b24 contig
62d19
300000
250000
200000
150000
100000
50000
0
100000
50000
0
(a)
(b)
(c)
0
50000
1000000
50000
100000
150000
200000
250000
300000
62d19
63n5-43b24 contig
