# Supplementary material for: Genomic complexity of the variable region-containing chitin-binding proteins in amphioxus
Source: BMC Genet. 2008 Dec 1;9:78. doi: 10.1186/1471-2156-9-78 (PMC2632668; doi:10.1186/1471-2156-9-78)
Supplement: Additional file 3 — Dot plot pairwise comparison of BAC 62d19 and BAC contig 63n5-43b24 exhibits discontinuities associated with allelic polymorphism, notably across the region encoding the VCBP2/5 cluster. [file 1471-2156-9-78-S3.pdf]

Additional file 3: Dot plot pairwise comparison (window size of 11) of 62d19 and contig 63n5-43b24 (haplotype B and A, respectively) exhibits discontinuities associated with allelic polymorphism, notably across the region encoding the VCBP2/5 cluster. Three repetitive regions [(a) – (c)] flank the VCBP gene cluster. Elements of repeats (b) and (c) are out of range for Bac 62d19 (see Additional file 2); the relative position of repeats (b) and (c) are marked vertically (reference to contig 63n5-43b24 on X-axis) to demonstrate sequence features of 62d19 found within those repeats. The second repeat (b) contains short alternating units (black triangles) derived from the first repeat (a). Short fragments of the third repeat (c) can also be found throughout 62d19 (grey triangles; see Additional file 1).

Figure S2

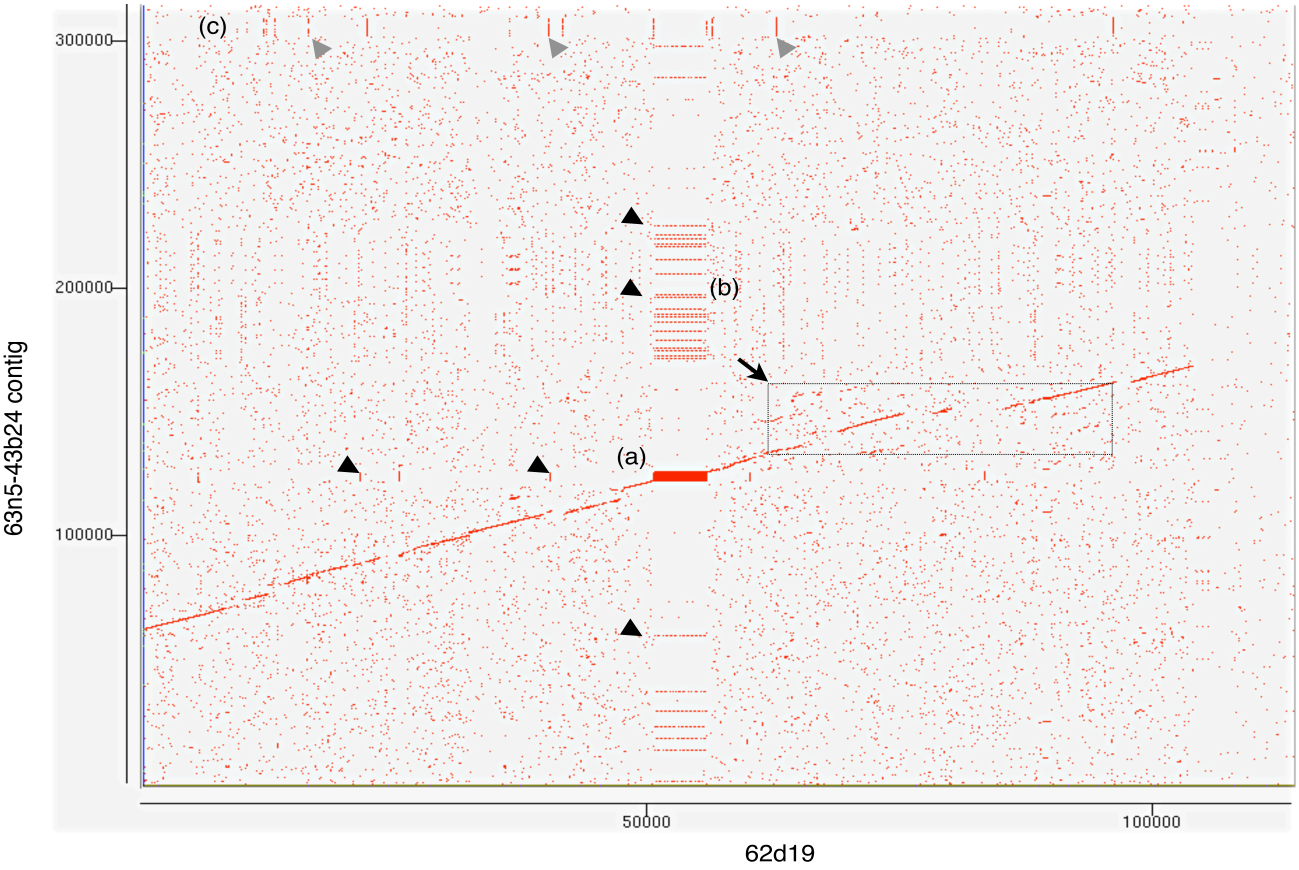

(c)
(b)
(a)
300000
200000
100000
63n5-43b24 contig
50000
100000
62d19
