# Supplementary material for: Genomic complexity of the variable region-containing chitin-binding proteins in amphioxus
Source: BMC Genet. 2008 Dec 1;9:78. doi: 10.1186/1471-2156-9-78 (PMC2632668; doi:10.1186/1471-2156-9-78)
Supplement: Additional file 4 — Dot plot pairwise comparison of the reverse complement of BAC 62d19 and BAC contig 63n5-43b24, and the corresponding region of the amphioxus genome. [file 1471-2156-9-78-S4.pdf]

Additional file 4. Dot plot pairwise comparison (window size of 11) of the reverse complement of BACs 62d19 and contig 63n5-43b24, and the corresponding region of the amphioxus genome (last 350 kb of scaffold_295 [native orientation] and the first 300 kb [reverse complement] of scaffold_82). Dot plot comparisons indicate that the contig formed by BACs 63n5 and 43b24 corresponds to the scaffold_82 allele and BAC 62d19 corresponds to the scaffold_295 allele. Repetitive elements [(a) – (c)] are polymorphic (length variable) across haplotypes. Note length of repeat (b) in panel 5 and 7 varies in size and reflects assembly artifacts across the repeat. Banding patterns that span panel 1, 4, 13, and 16 represent breaks (i.e., unresolved sequence) in scaffold assemblies.

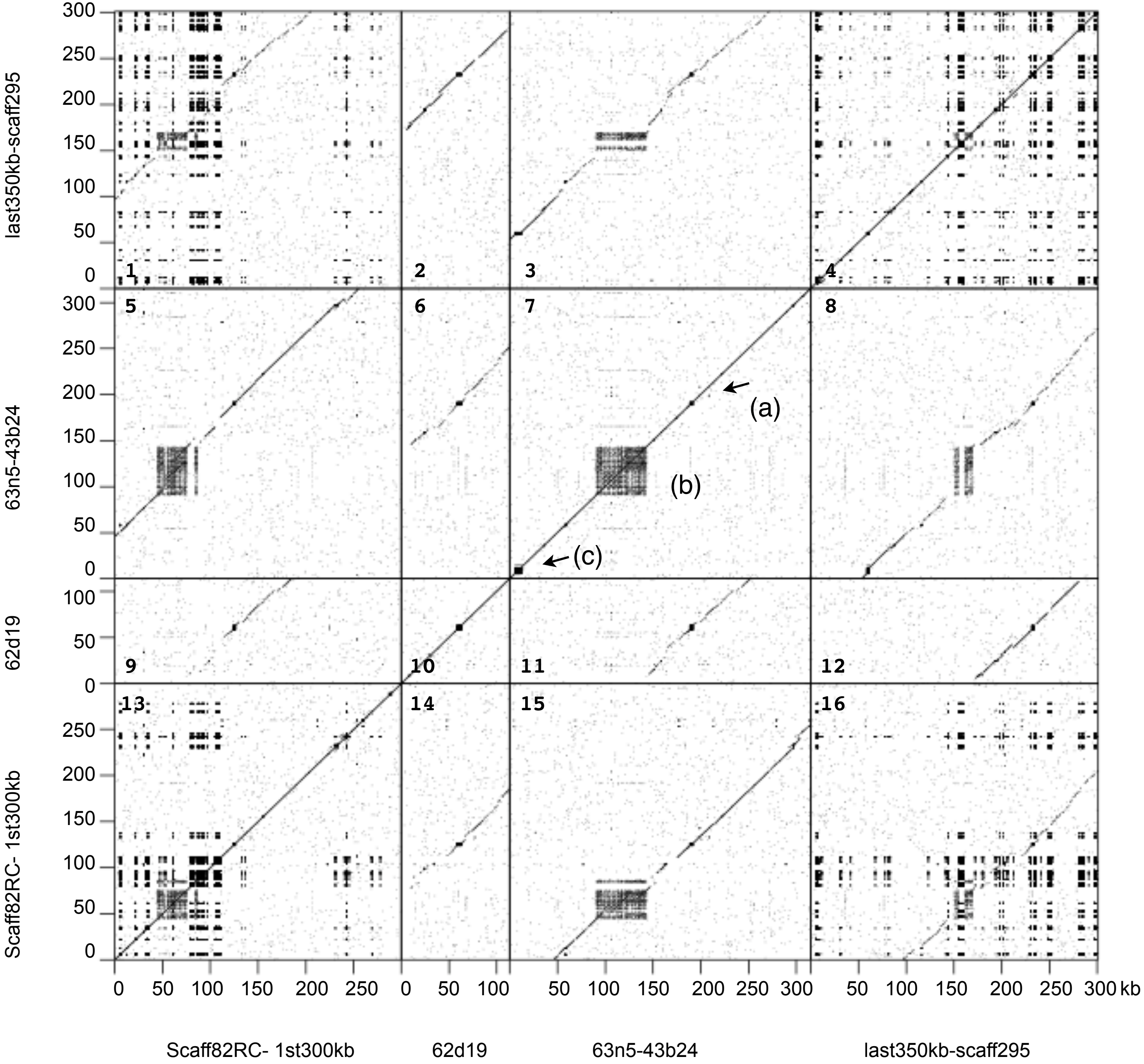

last350kb-scaff295
63n5-43b24
62d19
Scaff82RC- 1st300kb
1
2
3
4
5
6
7
8
9
10
11
12
13
14
15
16
(a)
(b)
(c)
0
50
100
150
200
250
300
kb
Scaff82RC- 1st300kb
62d19
63n5-43b24
last350kb-scaff295
