# Supplementary material for: Genomic complexity of the variable region-containing chitin-binding proteins in amphioxus
Source: BMC Genet. 2008 Dec 1;9:78. doi: 10.1186/1471-2156-9-78 (PMC2632668; doi:10.1186/1471-2156-9-78)
Supplement: Additional file 5 — Dot plot pairwise comparison of the reverse complement of the VCBP1/4-containing BAC 100j9 with the corresponding region of scaffold_295. [file 1471-2156-9-78-S5.pdf]

Additional file 5: Dot plot pairwise comparison (window size of 11) of the reverse complement of the VCBP1/4-containing BAC 100j9 with the corresponding region of scaffold_295 (bracketed region highlights duplicated genes), illustrates polymorphic variation across the alleles and corrects a Brafl1-specific misassembly across the VCBP1/4 region. A duplication of the VCBP1/4 gene pairs is evident in scaffold_295. Scaffold_869 (not shown, see Additional file 6) is the allelic counterpart represented by BAC 100j9 (haplotype A) but does not possess the VCBP1/4 gene pair. The first ~20 kb in 100j9 (boxed), as well as the second 1/4 gene pair, is displaced (i.e., assembly error) onto scaffold_295. BAC 100j9, which represents scaffold_869, allows the proper placement of the second pair of VCBP1/4 genes from scaffold_295 onto the corresponding allele. The paralogous duplication of VCBP1/4 genes on scaffold_295 is a result of misassembly. **(Authors note genome correction.)**

Figure S4

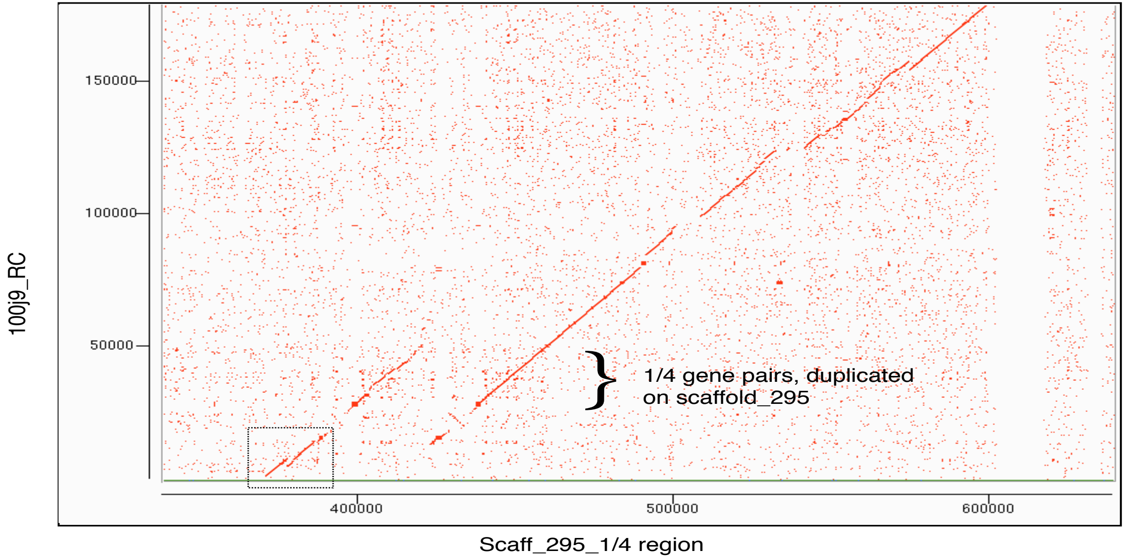
150000
100000
50000
400000
500000
600000
100j9_RC
Scaff_295_1/4 region
1/4 gene pairs, duplicated
on scaffold_295
