# Supplementary material for: Genomic complexity of the variable region-containing chitin-binding proteins in amphioxus
Source: BMC Genet. 2008 Dec 1;9:78. doi: 10.1186/1471-2156-9-78 (PMC2632668; doi:10.1186/1471-2156-9-78)
Supplement: Additional file 6 — Dot plot pairwise comparisons of scaffold_295 and scaffold_869 with the reverse complement of BAC 100j9 and PAC 34i7 (independent animal haplotype). [file 1471-2156-9-78-S6.pdf]

Additional file 6: Dot plot pairwise comparisons (window size of 11) of scaffold_295 and scaffold_869 with the reverse complement of BAC 100j9 and PAC 34i7 (allele from an independent animal). BAC 100j9 is most likely represented by scaffold_869, which is allelic to the corresponding VCBP1/4 region of scaffold_295, but lacks the VCBP1/4 genes (see Additional file 5). A large (~30 kb) haplotype-specific repetitive region is found at the end of scaffold_869 (see panel 10) and consists of alternating repetitive units, which are interspersed by exons exhibiting homology to tyrosine kinase-type receptors (possibly pseudogenes). Related repeating units (0.5 kb and larger) are distributed throughout scaffold_295 (panel 12). Several other repeating units of highly conserved sequence are distributed across these genetic regions, as well as throughout the genome. The allele represented by PAC 34i17 contains a large ~19 kb repeat (panel 7, which is unrelated to the repeat in scaffold_869) and varies extensively in length across alleles or could reflect an assembly artifact (in PAC 34i17). Segments of nearly identical sequence, ranging from ~0.5 to 3 kb, flank the VCBP1/4 region in both alleles of the reference genome.

Figure S5

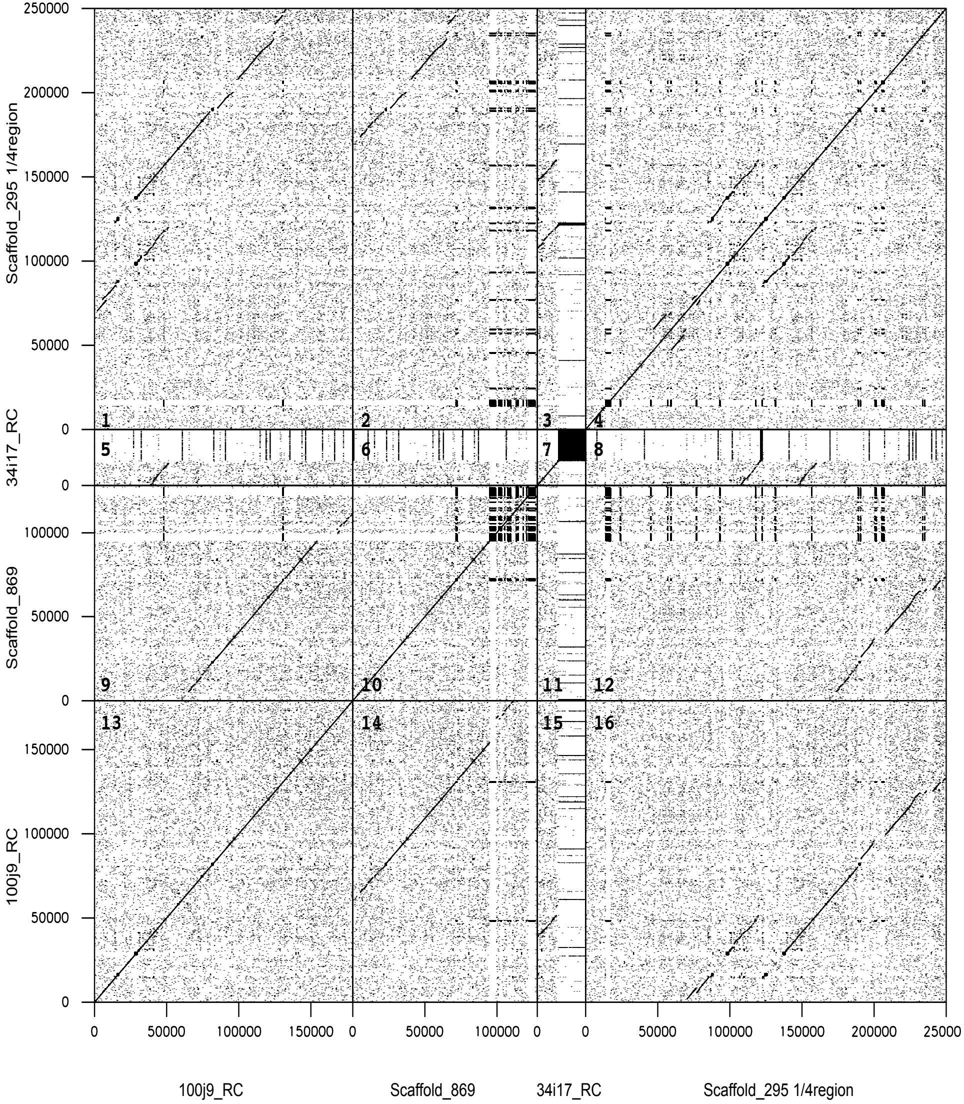

Scaffold_295 1/4region
34i17_RC
Scaffold_869
100j9_RC
100j9_RC
Scaffold_869
34i17_RC
Scaffold_295 1/4region
1
2
3
4
5
6
7
8
9
10
11
12
13
14
15
16
250000
200000
150000
100000
50000
0
