# Supplementary material for: Genomic complexity of the variable region-containing chitin-binding proteins in amphioxus
Source: BMC Genet. 2008 Dec 1;9:78. doi: 10.1186/1471-2156-9-78 (PMC2632668; doi:10.1186/1471-2156-9-78)
Supplement: Additional file 7 — Dot plot pairwise comparisons of the reverse complement of a ~100 kb region of scaffold_1 encoding VCBP3 with the corresponding region from BAC 90f15 and BAC 54h3, as well as PAC 30b18 (independent animal haplotype) encoding VCBP3. [file 1471-2156-9-78-S7.pdf]

Additional file 7. Dot plot pairwise comparisons (window size of 11) of the reverse complement of a ~100 kb region of scaffold_1 encoding VCBP3 with the corresponding region from BACs 90f15 and 54h3, as well as PAC 30b18 encoding VCBP3. A high degree of polymorphism across the genomic segments is evident. BAC 90f15 represents the genome allele (panel 6 and 11) from scaffold_1 and BAC 54h3 is the second allele not identified in the initial JGI assembly. Boxed area represents the VCBP3 genetic region in the corresponding area of scaffold_1 and PAC 30b18. **Authors note genome correction.**

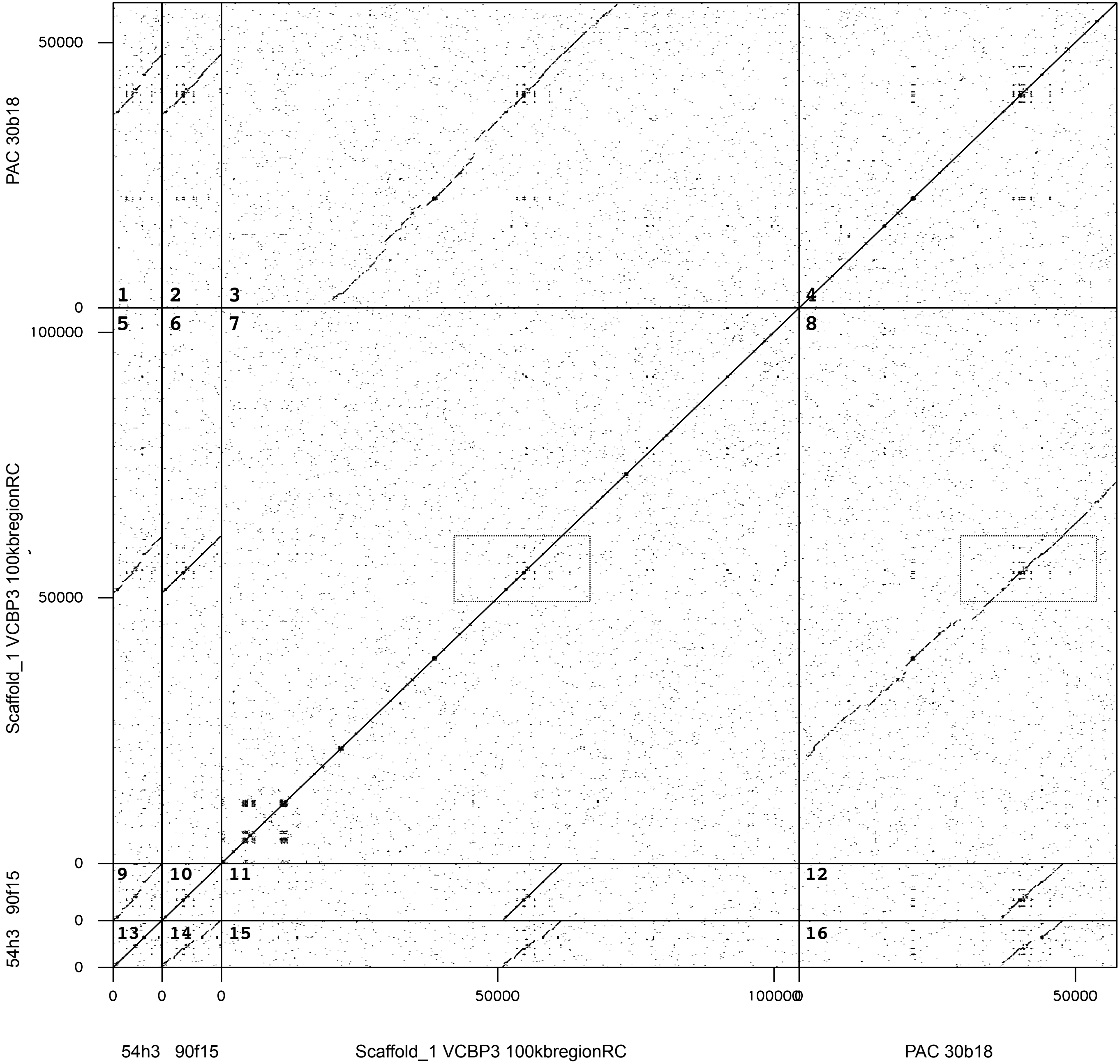
PAC 30b18
Scaffold_1 VCBP3 100kbregionRC
90f15
54h3
1
2
3
4
5
6
7
8
9
10
11
12
13
14
15
16
50000
0
100000
50000
0
0
0
0
0
0
50000
100000
0
50000
54h3
90f15
Scaffold_1 VCBP3 100kbregionRC
PAC 30b18
