# Supplementary material for: Genomic complexity of the variable region-containing chitin-binding proteins in amphioxus
Source: BMC Genet. 2008 Dec 1;9:78. doi: 10.1186/1471-2156-9-78 (PMC2632668; doi:10.1186/1471-2156-9-78)
Supplement: Additional file 8 — Dot plot comparisons of the VCBP3 gene region reveals that BAC 90f15 corresponds to genomic scaffold_1 (A) and that the other allele is highly polymorphic (B). [file 1471-2156-9-78-S8.pdf]

Additional file 8. Dot plot comparisons (window size of 11) of the VCBP3 gene region from upstream of the leader to just downstream of the final CBD exon reveals that BAC 90f15 corresponds to genomic scaffold_1 (A) and that the other allele is highly polymorphic (B).

A.

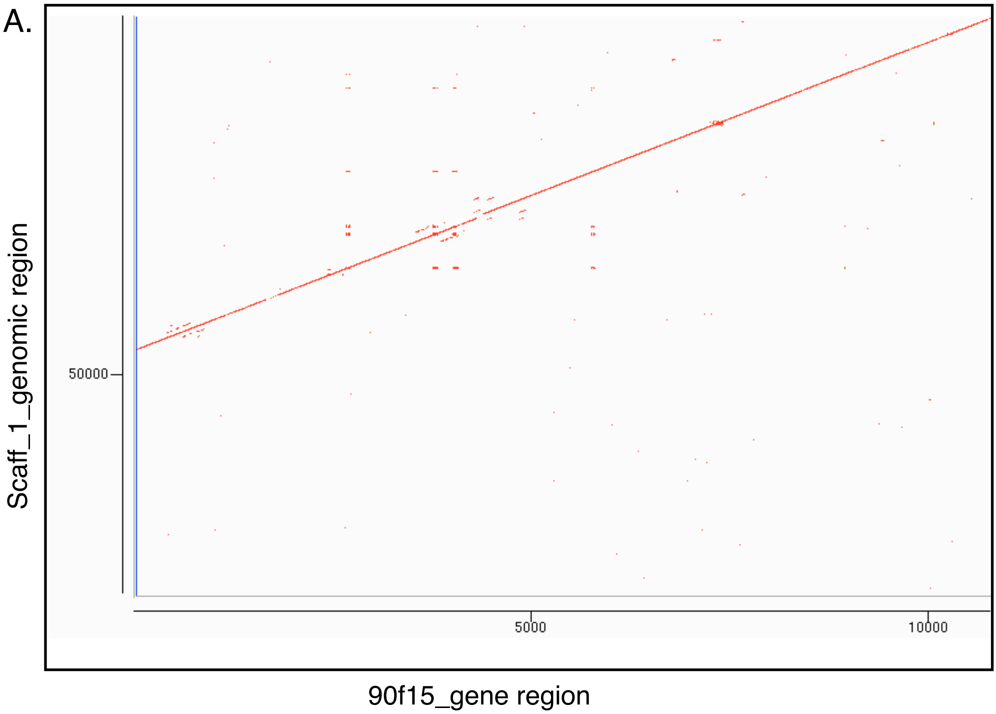

50000
5000
10000
Scaff_1_genomic region
90f15_gene region

B.

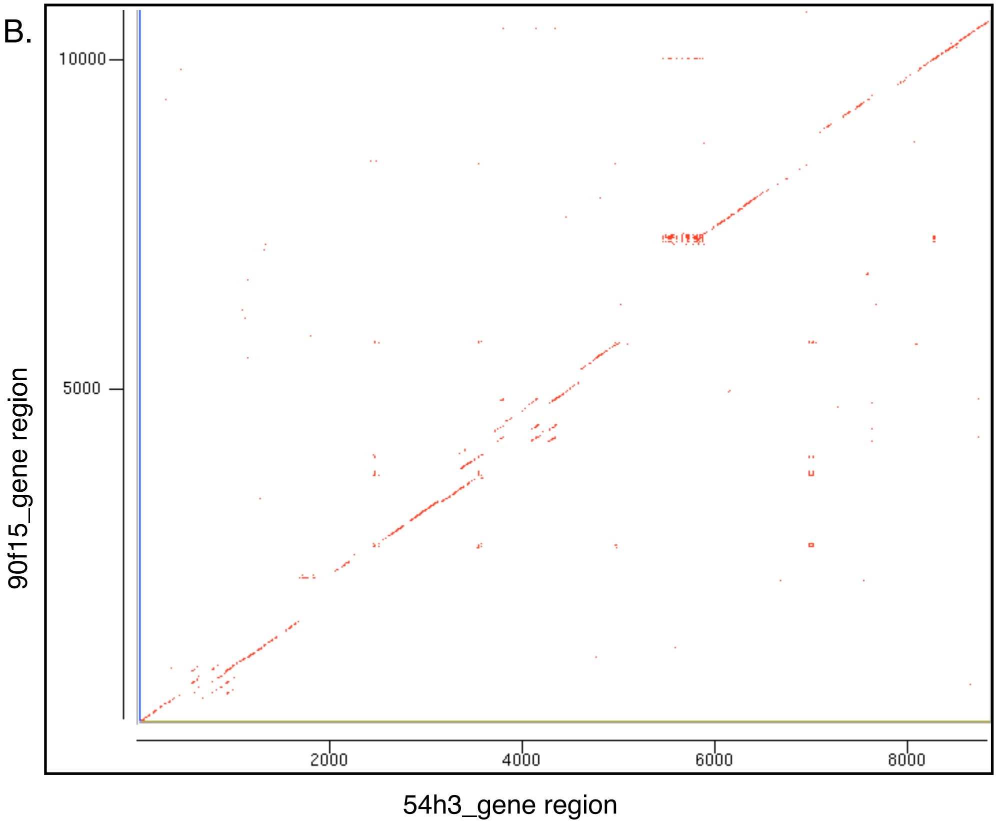

10000
5000
2000
4000
6000
8000
90f15_gene region
54h3_gene region
