# Supplementary material for: Genomic complexity of the variable region-containing chitin-binding proteins in amphioxus
Source: BMC Genet. 2008 Dec 1;9:78. doi: 10.1186/1471-2156-9-78 (PMC2632668; doi:10.1186/1471-2156-9-78)
Supplement: Additional file 9 — Genomic organization of the tyrosine recombinase domain-encoding retroelement found adjacent to the VCBP2/5 cluster. [file 1471-2156-9-78-S9.pdf]

Additional file 9. Genomic organization of the tyrosine recombinase domain-encoding retroelement found adjacent to the VCBP2/5 gene cluster. A. The gene consists of three main open reading frames, GAG-like, RT/RH –like, and tyrosine recombinase –like, on alternate reading frames. The gene is flanked by direct repeats (rectangles); duplication of direct repeats (red) are found upstream. B. Amino acid sequence homology with a Ngaro retrotransposon from *Lytechinus variegates* (green sea urchin); separated by domain.

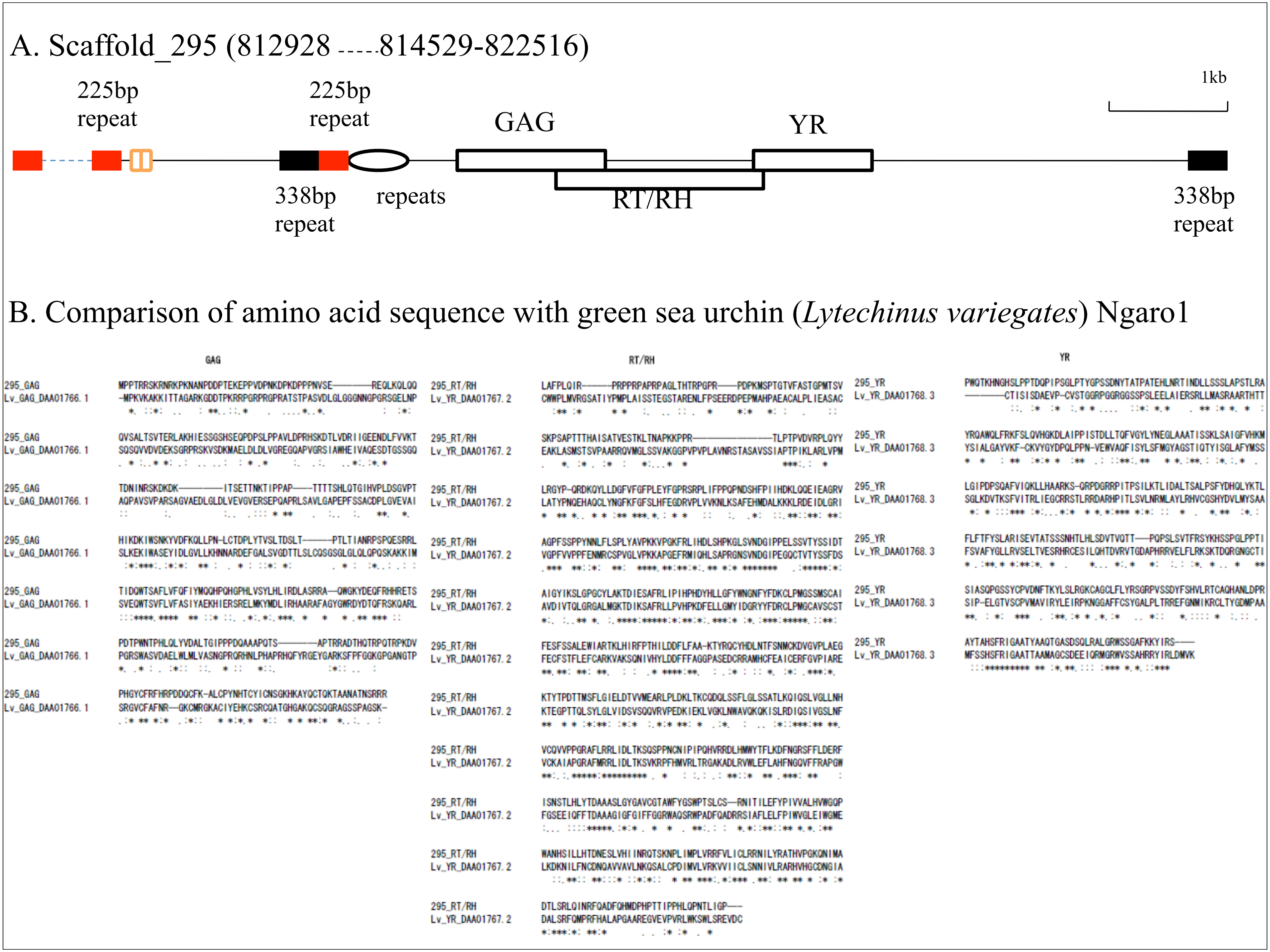
A. Scaffold_295 (812928 -----814529-822516)
225bp repeat
225bp repeat
GAG
YR
1kb
338bp repeat
repeats
RT/RH
338bp repeat
B. Comparison of amino acid sequence with green sea urchin (Lytechinus variegates) Ngaro1
GAG
RT/RH
YR
295_GAG
Lv_GAG_DAA01766.1
295_RT/RH
Lv_YR_DAA01767.2
295_YR
Lv_YR_DAA01768.3
