# Supplementary material for: Genomic complexity of the variable region-containing chitin-binding proteins in amphioxus
Source: BMC Genet. 2008 Dec 1;9:78. doi: 10.1186/1471-2156-9-78 (PMC2632668; doi:10.1186/1471-2156-9-78)

Additional file 10. Genomic profile (using the Gestalt viewer) across the BAC and PAC haplotype alleles described in this study. Included in the directional analyses are: repeat masking, inverted repeat finder (IRF: blue blocks represent the repeats, diagonal lines connect the corresponding repeats on opposite strands), open reading frames (ORFs), RNA fold predictions, %GC content and CpG prediction, as well as GenScan readouts. Please see Figure 3 legend of text for additional details on Gestalt viewer outputs. Haplotypic variation is evident across allelic regions (see Additional file 1 for BAC and PAC notation) and VCBP genes are annotated in yellow. A. BAC contig 63n5- 43b24; B. BAC 62d19; C. PAC 37d15; D. BAC 100j9; E. PAC 34i17; F. scaffold_1 spanning theVCBP3 region (showing ~100 kb span); G. PAC 30b18.

A. BAC contig 63n5-43b24

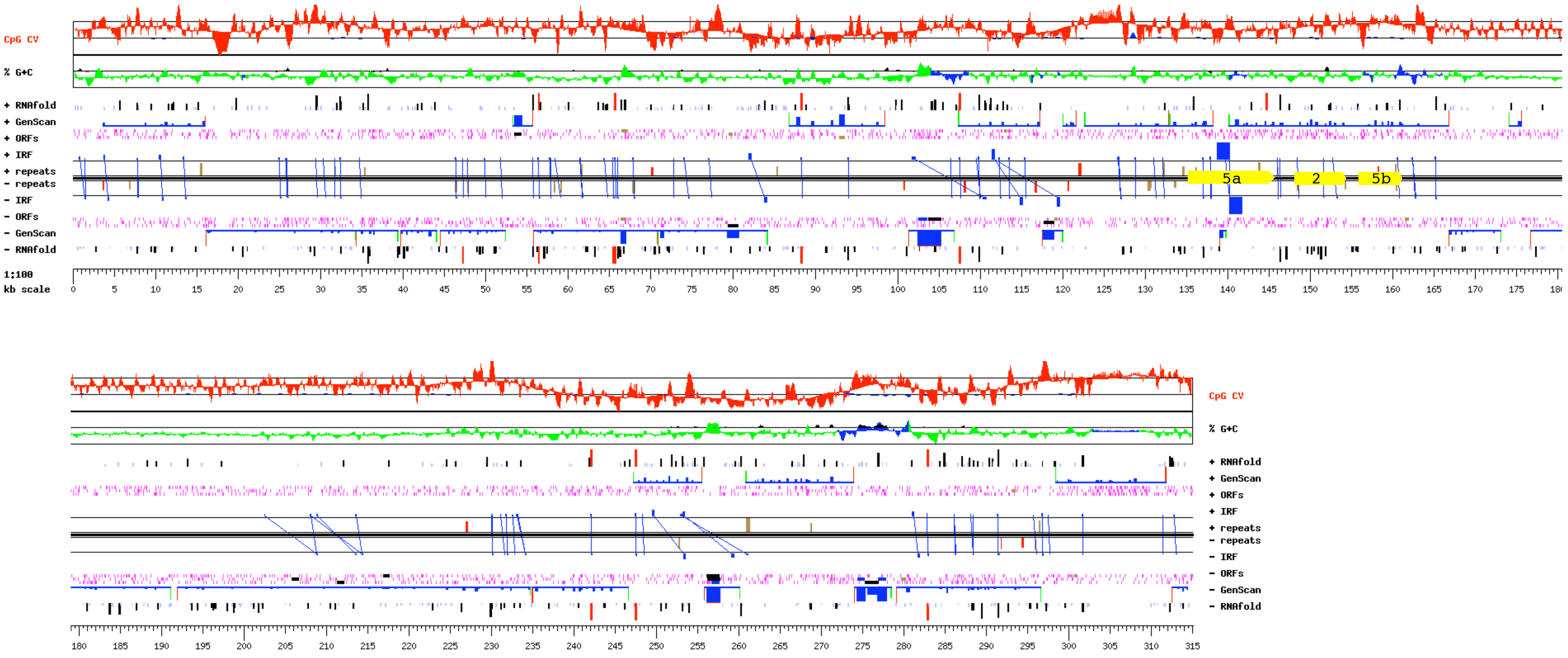

B. BAC 62d19

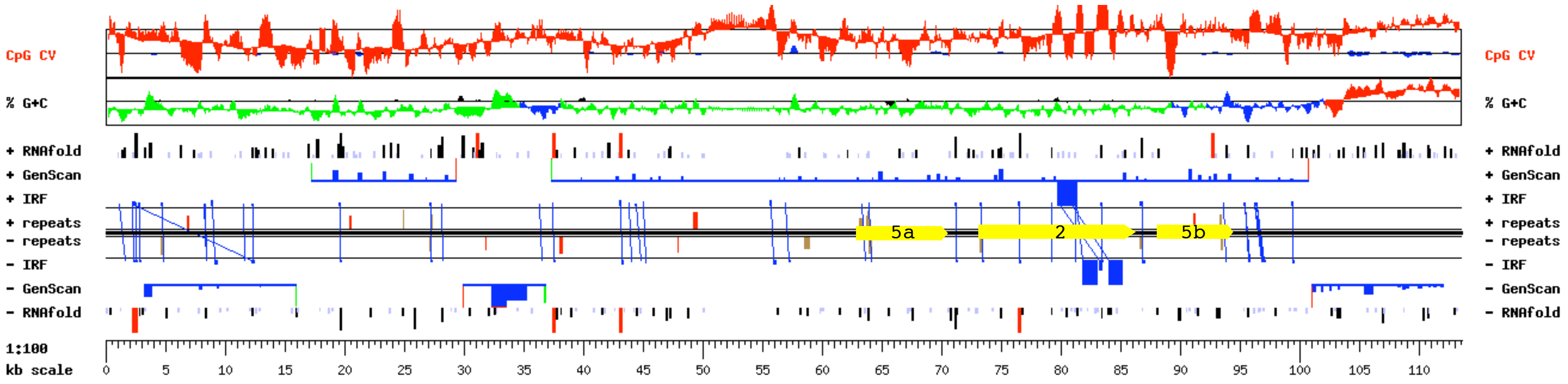

C. PAC 37d15

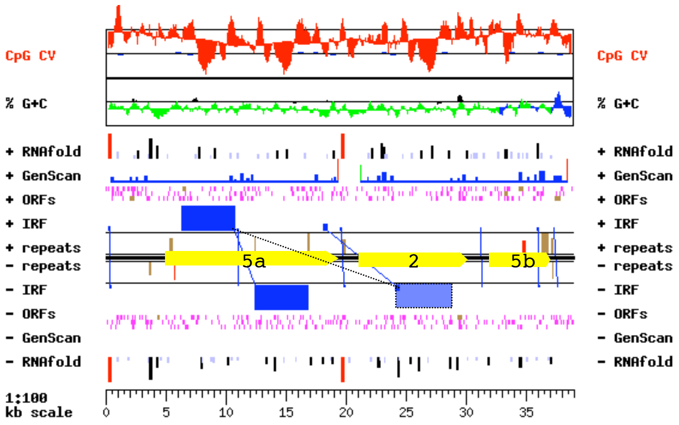

D. BAC 100j9

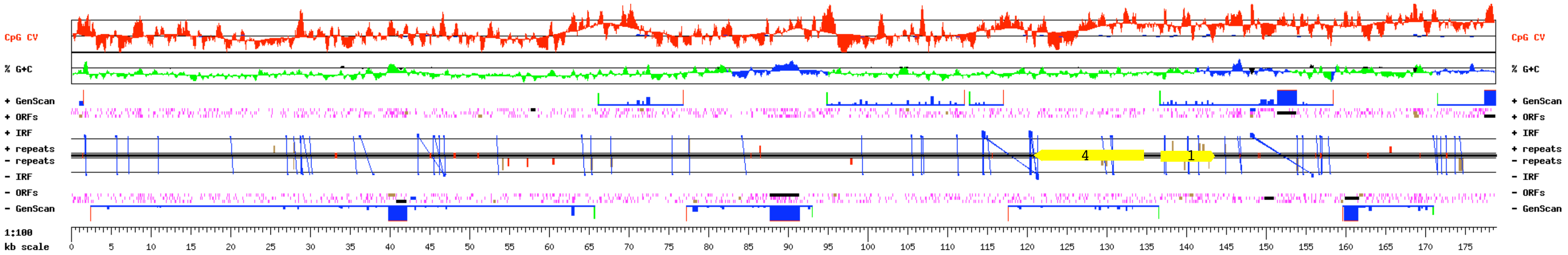

E. PAC 34i17

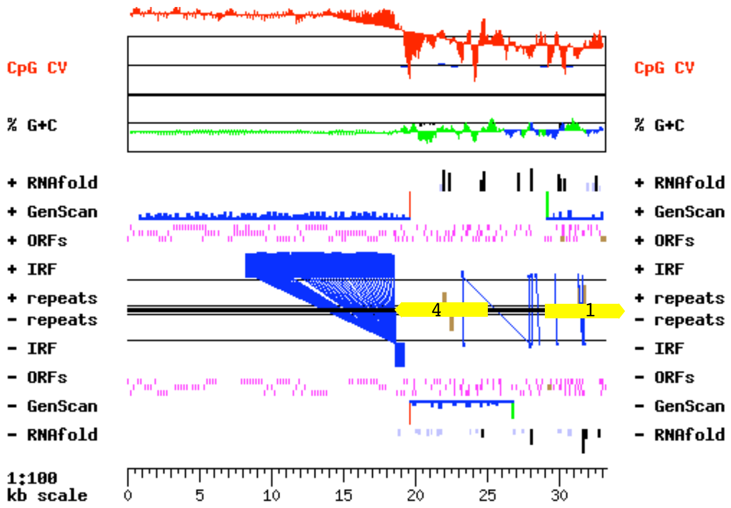

F. Scaffold_1 VCBP 3 region

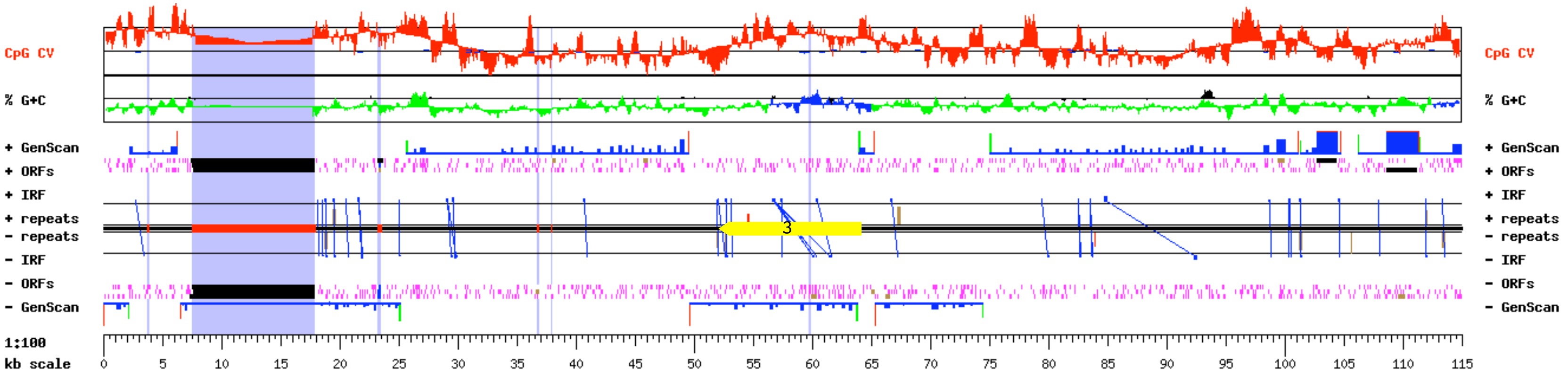

G. PAC 30b18

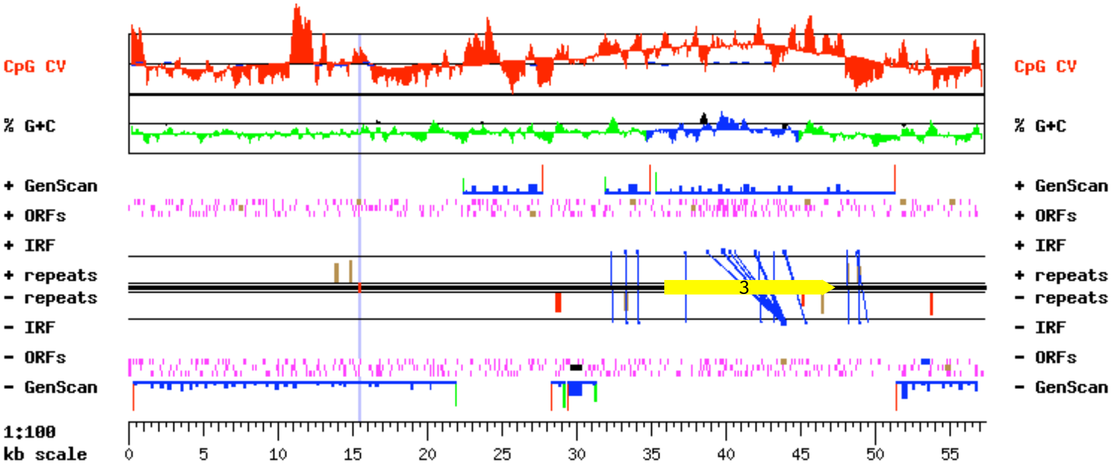

Supplement: Additional file 10 — Genomic representation (using the Gestalt viewer) across the BAC and PAC alleles described in this study. [file 1471-2156-9-78-S10.pdf]
